# Contributions of lipopolysaccharide and the type IVB secretion system to *Coxiella burnetii* vaccine efficacy and reactogenicity

**DOI:** 10.1038/s41541-021-00296-6

**Published:** 2021-03-19

**Authors:** Carrie M. Long, Paul A. Beare, Diane C. Cockrell, Jonathan Fintzi, Mahelat Tesfamariam, Carl I. Shaia, Robert A. Heinzen

**Affiliations:** 1 Coxiella Pathogenesis Section, Laboratory of Bacteriology, Division of Intramural Research, National Institute of Allergy and Infectious Diseases, National Institutes of Health, Hamilton, MT USA; 2 Biostatistics Research Branch, Division of Clinical Research, National Institute of Allergy and Infectious Diseases, National Institutes of Health, Rockville, MD USA; 3 Rocky Mountain Veterinary Branch, Division of Intramural Research, National Institute of Allergy and Infectious Diseases, National Institutes of Health, Hamilton, MT USA

**Keywords:** Bacteria, Bacterial genetics, Bacterial host response, Bacterial secretion, Bacterial techniques and applications

## Abstract

*Coxiella burnetii* is the bacterial causative agent of the zoonosis Q fever. The current human Q fever vaccine, Q-VAX^®^, is a fixed, whole cell vaccine (WCV) licensed solely for use in Australia. *C. burnetii* WCV administration is associated with a dermal hypersensitivity reaction in people with pre-existing immunity to *C. burnetii*, limiting wider use. Consequently, a less reactogenic vaccine is needed. Here, we investigated contributions of the *C. burnetii* Dot/Icm type IVB secretion system (T4BSS) and lipopolysaccharide (LPS) in protection and reactogenicity of fixed WCVs. A 32.5 kb region containing 23 *dot/icm* genes was deleted in the virulent Nine Mile phase I (NMI) strain and the resulting mutant was evaluated in guinea pig models of *C. burnetii* infection, vaccination-challenge, and post-vaccination hypersensitivity. The NMI ∆*dot/icm* strain was avirulent, protective as a WCV against a robust *C. burnetii* challenge, and displayed potentially altered reactogenicity compared to NMI. Nine Mile phase II (NMII) strains of *C. burnetii* that produce rough LPS, were similarly tested. NMI was significantly more protective than NMII as a WCV; however, both vaccines exhibited similar reactogenicity. Collectively, our results indicate that, like phase I LPS, the T4BSS is required for full virulence by *C. burnetii*. Conversely, unlike phase I LPS, the T4BSS is not required for vaccine-induced protection. LPS length does not appear to contribute to reactogenicity while the T4BSS may contribute to this response. NMI ∆*dot/icm* represents an avirulent phase I strain with full vaccine efficacy, illustrating the potential of genetically modified *C. burnetii* as improved WCVs.

## Introduction

*Coxiella burnetii* is a gram-negative, intracellular bacterium with near worldwide dissemination. This bacterium is the causative agent of the zoonosis Q fever. Human infection typically occurs following inhalation of infectious aerosols generated by domestic livestock reservoirs such as dairy cows, goats, and sheep. Human Q fever generally presents as an acute influenza-like illness although many individuals remain asymptomatic throughout infection^1^. Full recovery is common following acute illness, particularly after antibiotic treatment; however, some patients may develop persistent infections^2^ such as endocarditis, hepatitis, lymphadenitis, myocarditis, osteomyelitis, and/or vasculitis^1,3^ Because Q fever is potentially a debilitating disease and *C. burnetii* is environmentally stable and highly infectious by the aerosol route, the pathogen is considered a potential biological weapon and a U.S. Centers for Disease Control and Prevention (CDC), Division of Select Agents and Toxins (DSAT) select agent^4,5^.

A single subcutaneous dose of whole cell, inactivated *C. burnetii* has long been known as highly efficacious in human vaccination against Q fever^6,7^. Q-VAX^®^ is a whole cell vaccine (WCV) comprised of formalin-inactivated *C. burnetii* (Henzerling strain) that is licensed for use in Australia^8,9^. Here, it is administered primarily to at-risk populations, such as abattoir workers, and exhibits near 100% protective efficacy^10^. A potentially severe post-vaccination delayed-type hypersensitivity (DTH) skin response can occur at the inoculation site in individuals with pre-existing immunity due to previous symptomatic or asymptomatic infection^11^. This response necessitates extensive pre-screening of potential vaccinees by serologic and skin testing^11,12^. Accordingly, vaccine reactogenicity is a deterrent to the widespread use of *C. burnetii* WCVs in humans. A WCV derived from the Nine Mile strain, Coxevac^®^, has been approved for veterinary use in the European Union since 2015 and has been reported to cause injection site swelling in ruminants following repeated vaccination^13^.

Two surface exposed structures of *C. burnetii* that mediate host cell interactions and immune responses are the Dot/Icm type IVB secretion system (T4BSS) and lipopolysaccharide (LPS). The T4BSS delivers proteins with effector functions directly into the host cytosol, which mediates successful host cell infection^14^. *C. burnetii* contains two *dot/icm* loci; a major loci encoding 23 proteins and a minor loci encoding 2 proteins. Together these proteins form the surface exposed secretion apparatus^15^. Extensive structural modeling of the homologous Dot/Icm system of *Legionella pneumophila* reveals a macromolecular complex that includes several outer membrane lipoproteins^16,17^. Over 100 T4BSS substrates have been identified for *C. burnetii*, but only a few are functionally defined^18^. Most *dot/icm* mutants are incapable of replication within cultured cells^19–21^ and a *dotA* mutant exhibits attenuated virulence in *Galleria mellonella* larva^22^ and SCID mice^23^. The virulence potential of *dot/icm* mutants in an immunocompetent mammalian model is untested as is the relevance of the apparatus in vaccine-mediated immunity and hypersensitivity reactions.

Cumulative evidence suggests *C. burnetii* LPS enables pathogen avoidance of host immune detection by shielding the bacterial cell surface from binding by complement and antibodies and from recognition of toll-like receptor ligands, such as lipoproteins^24–26^. LPS has also been proposed to modulate intracellular trafficking of *C. burnetii* in macrophage host cells^27^. All natural *C. burnetii* isolates express full-length (phase I) LPS which is necessary for full virulence^28^. In fact, phase I LPS is currently the only virulence factor of *C. burnetii* defined in an immunocompetent animal model^29^. Serial in vitro passage of phase I *C. burnetii* in cultured cells, embryonated eggs, or synthetic medium results in LPS truncation. This process is known as phase variation and culminates in avirulent phase II organisms that synthesize LPS without *O*-antigen and several core sugars^29–34^. Shortly after the description of the phase variation phenomenon, Ormsbee et al.^35^ demonstrated in a guinea pig model that phase I organisms are significantly more potent as WCVs than phase II organisms, results repeated more recently in goat^36^ and mouse^37^ models. Thus, phase I LPS *O*-antigen is clearly a critical protective immunogen in vaccine-mediated immunity. Indeed, extracted phase I, but not phase II LPS, is protective in murine models^37,38^ with a caveat being the unknown degree of purity of these preparations. Similarly, an LPS peptide mimic vaccine elicits a protective antibody response in mice^39^. The role of LPS in vaccine reactogenicity has not been evaluated.

An improved Q fever vaccine should exhibit the potency of Q-VAX^®^ without the risk of a deleterious post-vaccination DTH response. Current and historic experimental vaccine platforms aimed at providing an efficacious, non-reactogenic Q fever vaccine include residue remaining after chloroform-methanol extraction of *C. burnetii*^40^, RNA-produced peptides containing predicted T-cell epitopes^41^, and protein subunit-adjuvant conjugates^42^. To the best of our knowledge, none of these strategies have yielded a vaccine with comparable efficacy to Q-VAX^®^. To achieve this goal, improved knowledge of both protective and DTH antigens, and the host immune response to these molecules, is needed. In the current study, we used guinea pig models to examine the roles of *C. burnetii* LPS and the T4BSS in virulence, vaccine-induced immunity, and the post-vaccination DTH response. This was achieved using isogenic *C. burnetii* strains, which express different LPS chemotypes^28^, and a mutant strain lacking the entire *dot/icm* apparatus.

## Results

### *C. burnetii* strain characterization

**Fig. 1:**
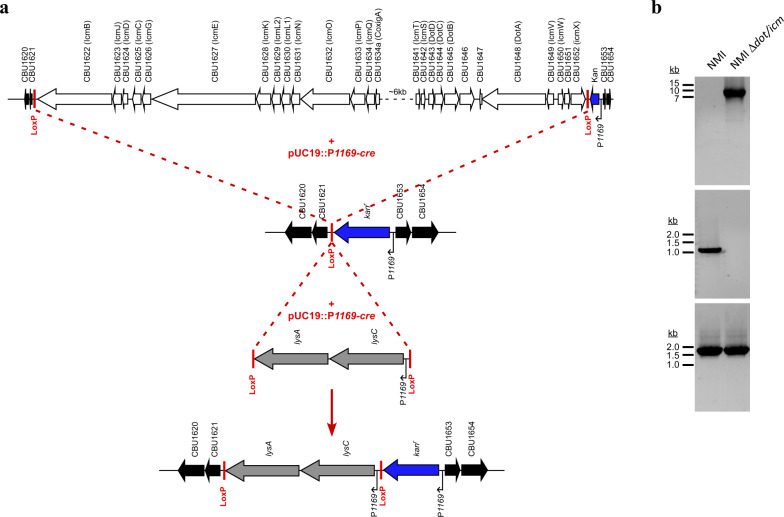
Construction of *C. burnetii* NMI ∆*dot/icm*. Cre-*lox* mediated deletion of the 32.5 kb *dot/icm* locus (*cbu1622*–*cbu1652*). **a** Schematic outlining the replacement of the *dot/icm* locus with *kan*^*r*^ and *lysCA* cassettes. A four-step Cre-*lox* strategy was used. Step 1 and 2 involved inserting *loxP* sites 5′ and 3′ of CBU1622 and CBU1652, respectively, using homologous recombination. Step 3 involved Cre mediated recombination between *loxP* sites resulting in deletion of the *dot/icm* locus. In step 4 Cre- mediated insertion of a *lysCA* cassette flanked by *loxP* sites into the single *loxP* site adjacent to *kan*^*r*^ was conducted to aid in cloning of the mutant. The location of *loxP* sites is denoted. **b** Agarose gels showing PCR product confirmation of deletion of the *dot/icm* locus and clonality of the mutant. The upper panel shows PCR products using primers flanking the *dot/icm* locus. NMI template DNA produced no band due to the size of the expected product (41.7 kb) while NMI Δ*dot/icm* template DNA produced a band of the expected size (9.1 kb). The middle panel depicts lack of amplification of CBU1628 via gene specific PCR of NMI Δ*dot/icm* DNA. The bottom panel shows amplification of a 1.6 kb region of gDNA that is conserved between NMI and NMI Δ*dot/icm*.

The *C. burnetii* strains Nine Mile RSA493 phase I (NMI), Nine Mile Crazy RSA514 (NMC), and Nine Mile RSA439 phase II, clone 4 (NMII) were utilized in these studies due to their historic experimental use, known virulence, and shared genetic background^29,30,43^. These isogenic strains are genetically similar with the exception of large genomic deletions of LPS biosynthesis genes in NMC and NMII^44^. NMI, NMC, and NMII strains are characterized by their production of full-length (phase I), intermediate length, and truncated (phase II) LPS, respectively^30^. A NMI RSA493 ∆*dot/icm* mutant was constructed using Cre-*lox*-mediated recombination to delete a 32.5 kb region containg 23 genes (*cbu1622*-*cbu1652*) encoding Dot/Icm apparatus proteins (Fig. 1a, b).

**Fig. 2:**
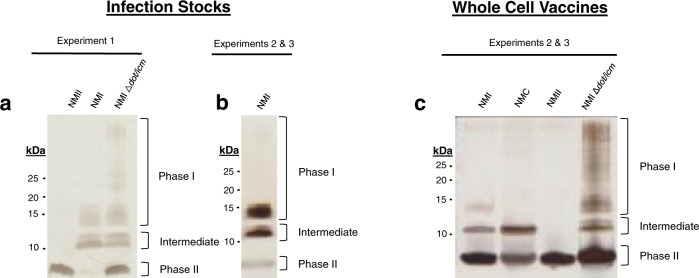
LPS profiles of *C. burnetii* strains by silver stain. The LPS profiles of *C. burnetii* strains used in this study were examined by silver stain. LPS of live infection stocks used in experiments 1 **a** and 2 & 3 **b**, along with whole cell vaccines used in experiments 2 & 3 **c** are displayed. Silver stain bands corresponding to phase I, intermediate, and phase II LPS, are indicated to the right of each gel while the marker sizes (kDa) are indicated to the left.

Three studies were conducted using guinea pig models to examine the influence of LPS length and the T4BSS in *C. burnetii* virulence, vaccine efficacy, and post-vaccination hypersensitivity. The experimental designs are outlined in Table 1. As LPS length is an important determinant of virulence and vaccine efficacy^29^, the LPS content of all *C. burnetii* stocks used for infections or as WCVs were characterized by silver stain (Fig. 2a–c) and immunoblot using antibodies specific for phase I, intermediate, and phase II LPS^30^ (Supplementary Fig. 1). NMI and NMII stocks used for infections in experiment 1 (virulence) displayed prototypical LPS molecules. Specifically, NMI expressed intermediate (~11 kDa; lipid A, inner and outer core sugars, and one repeating *O*-antigen unit lacking virenose) and full-length phase I LPS (>12 kDa; lipid A, all core sugars, and repeating *O*-antigen subunits) and NMII expressed only phase II LPS (~3 kDa; lipid A and inner core sugars) (Fig. 2a, b and Supplementary Fig. 1a–c)^30^. A low amount of phase II LPS was also produced by NMI which is reflective of low in vitro passage. Repeated in vitro passage in synthetic medium is required to generate clonal *C. burnetii* mutants. Consequently, the engineered NMI ∆*dot/icm* mutant appeared to express more phase II LPS than the NMI parent strain (Fig. 2a and Supplementary Fig. 1 a–c). In experiments 2 (vaccine efficacy) and 3 (post-vaccination hypersensitivity), both fixed and live *C. burnetii* strains were utilized. The live NMI stock used for infection displayed slightly more phase II LPS then the NMI used in experiment 1 (Fig. 2b and Supplementary Figs. 1d–f). For WCV stocks, NMI and NMC were passaged in vitro prior to fixation to promote the formation of phase II LPS equivalent to NMI ∆*dot/icm* (Fig. 2c and Supplementary Fig. 1i). NMC WCV expressed prototypical intermediate (~11 kDa) LPS molecules (Fig. 2c).

**Table 1:** Experimental descriptions.

*Experiment 1—Virulence*		
Infection > 14 Days > Euthanasia		
Infection strain		Sample size
Saline		4
^a^NMI		4
^b^NMII		4
^c^NMI Δ*dot/icm*		4
*Experiment 2 – Vaccine Efficacy*		
Vaccination > 28 Days > Infection > 14 Days > Euthanasia		
Vaccine strain	Infection strain	Sample size
Saline	Saline	3
Saline	NMI	4
NMI	NMI	4
Q-VAX^®^	NMI	4
NMII	NMI	4
NMI Δ*dot/icm*	NMI	4
*Experiment 3—Post-vaccination hypersensitivity*		
Infection > 42 Days > Skin Test > 21 Days > Euthanasia		
Infection strain	Skin test strain	Sample size
Saline	Saline	4
Saline	NMI	4
NMI	Saline	4
NMI	NMI	4
NMI	^d^NMC	4
NMI	NMII	4
NMI	NMI ?*dot/icm*	4

^a^NMI: Nine Mile I RSA493

^b^NMII: Nine Mile II RSA439

^c^NMI ∆*dot/icm*: Nine Mile I RSA493 with *dot/icm* deletion

^d^NMC: Nine Mile Crazy RSA514

### NMI ∆*dot*/*icm* is avirulent in a guinea pig model of Q fever

**Fig. 3:**
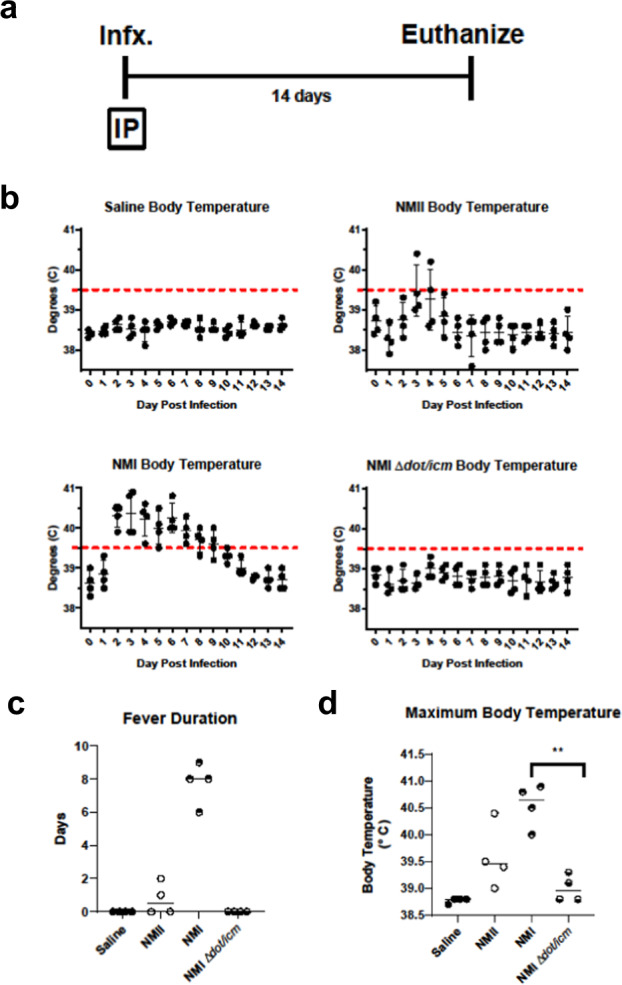
Infection by NMI ∆*dot/icm* does not cause fever in guinea pigs. **a** Body temperatures were recorded daily from the day of infection (day 0) until the day of euthanasia (day 14). **b** Temperatures were plotted for each individual animal per time point throughout the duration of infection. Fever was defined as ≥39.5 °C and is indicated by the dotted line. Fever duration **c** and maximum body temperature at any given point post-infection **d** are presented for each animal. These two endpoints were evaluated based on differences in group means (horizontal bar) with associated confidence intervals. Error bars represent standard deviation. For each reported comparison, *p*-values were computed at the α = 0.05 level for two-sided two-sample *t* tests, allowing for unequal variances between groups. No adjustment was made for multiple comparisons due to the sample sizes (*n* = 4) and the descriptive nature of the study. **p ≤ 0.01.

We first evaluated the virulence of NMI ∆*dot/icm* in guinea pigs (Table 1, experiment 1). Animals were intraperitoneally infected with 10^6^ genome equivalents (GE) of *C. burnetii* (NMII, NMI, or NMI ∆*dot/icm*) or saline (Fig. 3a). Body temperatures were recorded daily for 14 days following infection (Fig. 3b). As expected, saline-treated negative control animals remained afebrile (body temperature of ≤39.5 °C) and maintained a consistent body temperature throughout the duration of each study. Conversely, animals from the NMII and NMI-infected groups developed fever of varied duration and magnitude (Fig. 3c). Two of the NMII-infected animals developed fever, one for a single day and the other for two days. All NMI-infected animals developed fever which lasted for 6–9 days. In contrast, NMI ∆*dot/icm*-infected animals remained afebrile throughout the duration of the study. On average, NMI ∆*dot/icm*-infected animals experienced significantly lower maximum body temperatures than NMI-infected animals (Fig. 3d).

**Fig. 4:**
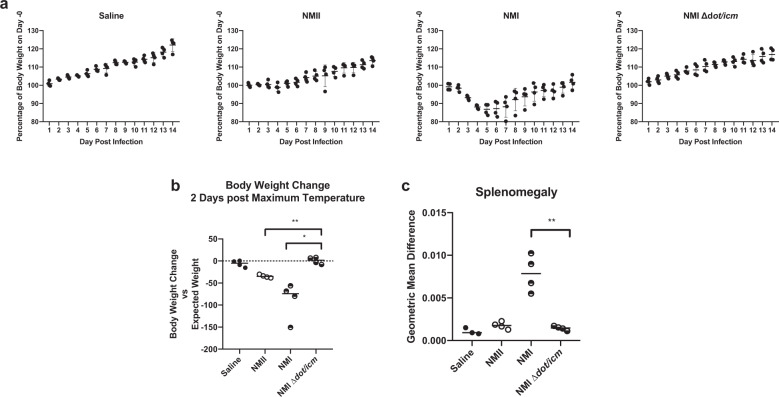
Infection by NMI ∆*dot/icm* does not cause body weight loss or splenomegaly in guinea pigs. **a** Body weight change is presented as percentage of body weight altered at any given day compared to day 0 post-infection. **b** Body weight change at 2 days post maximum temperature is presented as body weight change vs expected weight at the measured time point for each individual animal, with a dotted line at 0 indicating no change. **c** At euthanasia (14 days post-infection), splenomegaly was quantified and data are presented as geometric mean difference of spleen weight normalized to total body weight. Body weight change and splenomegaly were evaluated based on differences in group means (horizontal bar) with associated confidence intervals. Error bars represent standard deviation. For each reported comparison, *p*-values were computed at the α = 0.05 level for two-sided two-sample *t* tests, allowing for unequal variances between groups. No adjustment was made for multiple comparisons due to the sample sizes (*n* = 4) and the descriptive nature of the study. *p ≤ 0.05, **p ≤ 0.01.

Body weight measurements over 14 days mirrored body temperatures both in magnitude and timing (Figs. 3b and 4a). The expected growth trajectory in the absence of intervention was estimated by linear regression fit via generalized estimating equations to body weight measurements taken on animals in the saline group. Body weight alterations relative to the expected weight were analyzed two days after the day of maximum body temperature for each animal as this was found to coincide with the day of maximum weight loss. NMI ∆*dot/icm*-infected animals exhibited similar body weight alteration kinetics to the saline mock-infection group (Fig. 4a). These two groups did not experience any statistically significant mean differences in weight relative to their baseline trajectory at two days post maximum temperature (Fig. 4b). In contrast, NMI and NMII-infected animals lost body weight during the study, beginning around day 3 post-infection. Both groups exhibited statistically significant decreases in average body weight relative to their baseline trajectory at two days post maximum body temperature compared to NMI ∆*dot/icm*-infected animals (Fig. 4b).

Splenomegaly, another endpoint related to *C. burnetii*-induced disease progression in guinea pigs^45^, was measured following euthanasia. As a percentage of body weight, geometric mean spleen weights were comparable in saline, NMII, and NMI ∆*dot/icm*-infected animals (Fig. 4c). In contrast, geometric mean spleen weights were significantly increased in the NMI-infected animals compared to NMI ∆*dot/icm*-infected animals (Fig. 4c).

### NMI ∆*dot/icm* WCV protects against *C. burnetii* challenge in a guinea pig model of Q fever

**Fig. 5:**
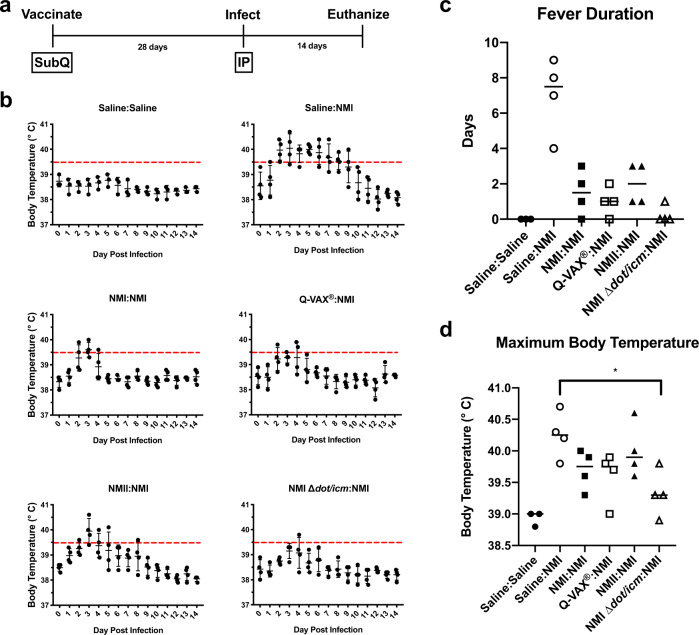
NMI ∆*dot/icm* WCV protects against *C. burnetii* challenge. **a** Guinea pigs were subcutaneously vaccinated with 25 μg of *C. burnetii* WCV or Q-VAX^®^, or mock infected with saline, then allowed to rest for 28 days. Animals were then intraperitoneally infected with 10^6^ GE NMI or mock infected with saline, monitored for 14 days, and euthanized. **b** Body temperatures were recorded daily from the day of infection (day 0) until the day of euthanasia (day 14). Temperatures were plotted for each animal per time point throughout the duration of infection. Fever was defined as ≥39.5 °C and is indicated by the dotted line. Fever duration **c** and maximum body temperature **d** are presented for each animal. These two endpoints were evaluated based on differences in group means (horizontal bar) with associated confidence intervals. For each reported comparison, *p*-values were computed at the α = 0.05 level for two-sided two-sample *t* tests, allowing for unequal variances between groups. No adjustment was made for multiple comparisons due to the sample sizes (*n* = 4 except for Saline:Saline with *n* = 3) and the descriptive nature of the study. *p ≤ 0.05.

Next, we evaluated the efficacy of NMI ∆*dot/icm* as a WCV (Table 1, experiment 2). Guinea pigs were subcutaneously vaccinated with 25 μg of paraformaldehyde fixed NMI, NMII, or NMI ∆*dot/icm*. Q-VAX^®^ (25 μg) and saline were used as positive and negative vaccination controls, respectively. Over a 28-day rest period, no alterations in body temperature or weight were observed as a result of vaccination (data not shown). Animals were then intraperitoneally challenged with 10^6^ genomic equivalents (GE) of *C. burnetii* NMI, or mock challenged with saline (Fig. 5a). Body temperatures were recorded daily for 14 days following infection and are presented in Fig. 5b. Mock vaccinated and mock challenged controls (Saline:Saline) did not exhibit fever or significant changes in body temperature for the duration of the monitoring period. All saline-mock vaccinated and NMI-challenged animals (Saline:NMI) developed sustained fevers (4–9 days in duration) by day 3 post-infection (Fig. 5b, c). Guinea pigs in NMI:NMI and Q-VAX^®^:NMI (vaccination:challenge) groups exhibited similar body temperature responses with low grade fevers occurring at 2 to 4 days post-infection for several animals. All NMII:NMI animals experienced fever, with an average duration of 2 days. Notably, most NMI ∆*dot/icm*:NMI animals did not experience fever, the exception being a single animal with a minimal fever at 4 days post-infection (temp = 39.8 °C). NMI ∆*dot/icm* was the only vaccination treatment that resulted in significantly decreased maximum body temperature compared to unvaccinated, NMI-challenged control animals (Fig. 5d).

**Fig. 6:**
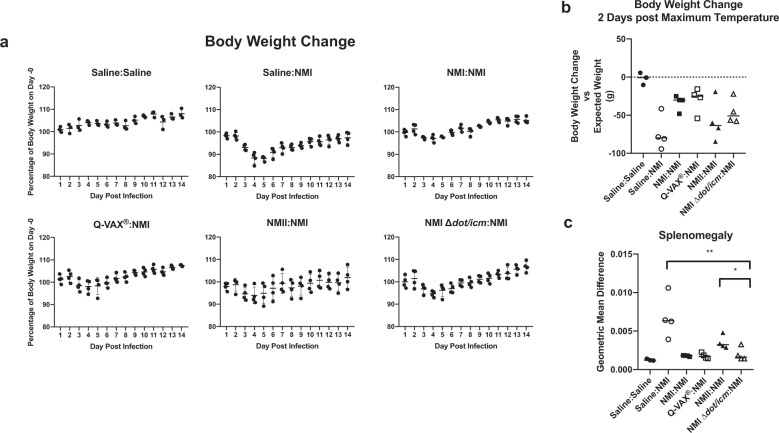
NMI ∆*dot/icm* WCV protects guinea pigs against body weight loss and splenomegaly following *C. burnetii* challenge. **a**. Guinea pigs were subcutaneously vaccinated with 25 μg of *C. burnetii* WCVor Q-VAX^®^, or mock infected with saline, then allowed to rest for 28 days. Animals were then intraperitoneally infected with 10^6^ GE NMI or mock infected with saline, monitored for 14 days, and euthanized. Body weight change is presented as percentage of body weight gained/lost at any given day compared to day 0 post-infection. **b** Body weight change at 2 days post maximum temperature is presented as body weight change versus expected weight at the measured time point for each individual animal, with a black dotted line at 0, indicating no change. **c** At euthanasia (14 days post-infection), splenomegaly was quantified and data are presented as geometric mean difference of spleen weight normalized to total body weight. Body weight change and splenomegaly were evaluated based on differences in group means (horizontal bar) with associated confidence intervals. Error bars represent standard deviation. For each reported comparison, *p*-values were computed at the α = 0.05 level for two-sided two-sample *t* tests, allowing for unequal variances between groups. No adjustment was made for multiple comparisons due to the sample sizes (*n* = 4 except for Saline:Saline with *n* = 3) and the descriptive nature of the study. *p ≤ 0.05 and **p ≤ 0.01.

Body weight measurements over 14 days mirrored body temperatures both in magnitude and timing (Figs. 5b and 6a). Saline:Saline animals exhibited stable body weight kinetics and we used measurements from this group to estimate the expected weight trajectory in the absence of intervention. All NMI-challenged animals lost body weight beginning at day 3 post-infection, regardless of vaccination status. Saline:NMI animals lost the most body weight relative to their baseline trajectory at two days post maximum temperature compared to all other vaccinated and challenged groups, with an average loss of 73.9 g (Fig. 6b). Among vaccinated and challenged groups, NMII:NMI lost the most body weight two days post maximum body temperature, with an average loss of 57.7 g. NMI ∆*dot/icm* vaccine efficacy was further illustrated by NMI ∆*dot/icm*:NMI animals demonstrating significantly less splenomegaly than Saline:NMI and NMII:NMI groups at 14 days post-infection (Fig. 6c). Lower overall estimated splenic bacterial burdens were observed in all vaccinated animals compared to that of Saline:NMI unvaccinated controls (Supplementary Fig. 2).

Two mesenteric lymph nodes (mLN) were excised, counted (Supplementary Fig. 3a), and analyzed for lymphocyte marker expression (Supplementary Fig. 3b–d) at 14 days post challenge. mLN counts were significantly decreased in NMI ∆*dot/icm*:NMI animals compared to Saline:NMI animals. Additionally, CD8^+^ T-cell median fluorescence intensity (MFI) was significantly decreased in NMI ∆*dot/icm*:NMI animals compared to Saline:NMI animals.

### WCV skin testing reveals contributions of the T4BSS of *C. burnetii* to the post-vaccination hypersensitivity response

**Fig. 7:**
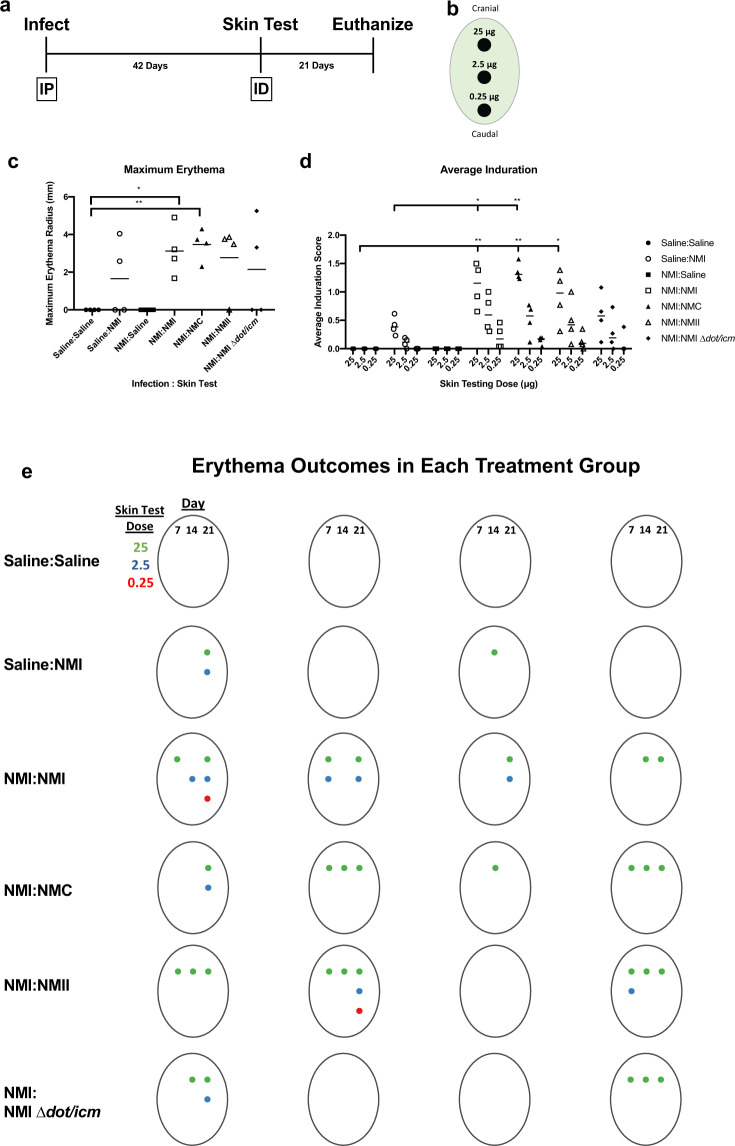
Skin testing reveals the contribution of LPS and the T4BSS of *C. burnetii* in post-vaccination hypersensitivity. **a** Animals were infected intraperitoneally (IP) with 10^6^ NMI, or mock infected with saline, allowed to rest for 42 days, then intradermally (ID) injected (skin tested) with 25, 2.5, and 0.25 μg fixed *C. burnetii* or saline **b** Animals were then monitored for 21 days and euthanized. Erythema was measured at each skin testing site and is represented by maximum erythema radius (mm) at any day or skin testing dose **c** Average induration over the entire monitoring period is presented in (**d**). Overall erythema outcomes for each animal in each treatment group is presented in (**e**); both day of measurement (columns) and skin testing site dose (row) are represented. A colored dot represents measurable erythema at the corresponding day post-skin test and skin test lesion site. Maximum erythema and average induration were evaluated based on differences in group means (horizontal bar) with associated confidence intervals. For each reported comparison, *p*-values were computed at the α = 0.05 level for two-sided two-sample *t* tests, allowing for unequal variances between groups. No adjustment was made for multiple comparisons due to the sample sizes (*n* = 4) and the descriptive nature of the study. *p ≤ 0.05 and **p ≤ 0.01.

Skin testing (Table 1, Experiment 3) was initiated by intraperitoneally infecting guinea pigs with 10^6^ NMI to allow for immune sensitization. All NMI-infected animals displayed fever and weight loss at similar levels for 14 days following infection, confirming successful infection (Supplementary Fig. 4). At 42 days post-infection, animals were intradermally injected with 25, 2.5, and 0.25 μg of fixed *C. burnetii* or saline at three sites in each animal’s flank for hypersensitivity skin testing (Fig. 7a, b). For 21 days following skin testing no changes were observed in body temperature or weight (Supplementary Fig. 5) and no changes were observed in mesenteric lymph node (mLN) size at euthanasia (Supplementary Fig. 6a) among experimental animals. At 21 days post-skin testing, splenomegaly was observed in NMI-infected and skin tested groups but not the uninfected, NMI-skin tested group (Supplementary Fig. 6b).

Erythema and induration were measured for 21 days following skin testing and are reported as erythema area (Fig. 7c and Supplementary Fig. 7) and induration severity (Fig. 7d and Supplementary Fig. 8) at the injection site. A visual representation of erythema outcomes is presented in Fig. 7e. As expected, neither Saline:Saline nor NMI:Saline animals exhibited measurable erythema or induration throughout the monitoring period. Half of the non-sensitized, NMI-skin tested control animals (Saline:NMI) exhibited measurable erythema at the 25 μg skin testing site beginning at day 14 or 21. Minimal induration was observed in all of the animals in this group at the 25 μg skin testing site. All sensitized and *C. burnetii* skin tested groups exhibited more severe responses than non-sensitized groups. NMI:NMI, NMI:NMC, and NMI:NMII groups exhibited similar kinetics and magnitude of response, beginning with measurable erythema at day 7 at the 25 μg site for animals from all three groups and at the 2.5 μg site for NMI:NMI and NMI:NMII animals. NMI:NMI and NMI:NMC groups exhibited statistically significant increases in maximum erythema at any given skin testing site compared to the Saline:Saline group. Induration at the 25 μg site was initially detected at day 2 in half of the NMI:NMC animals and at day 4 in 3/4 of NMI:NMI animals and half of NMI:NMII animals. Induration was sustained for the remainder of the 21 day post-skin test period and was observed at additional skin testing sites (2.5 and 0.25 μg) in animals from NMI:NMI, NMI:NMC, and NMI:NMII groups. Average induration at the 25 μg skin testing site was significantly increased for these groups compared to Saline:Saline negative controls and for NMI:NMI and NMI:NMC groups compared to unsensitized Saline:NMI controls. Erythema and induration were observed at 0.25 μg sites for animals in both NMI:NMI and NMI:NMII groups, emphasizing the relative intensity of these responses. In contrast, NMI:NMI ∆*dot/icm* animals demonstrated erythema at the 25 and 2.5 μg sites, but not at the 0.25 μg site, and only experienced induration at the 0.25 μg site transiently. Maximum erythema was similar among all sensitized and *C. burnetii* skin tested groups, with half of the NMI:NMI ∆*dot/icm* animals displaying values comparable to all other Nine Mile strain skin tested groups. The remaining two NMI:NMI ∆*dot/icm* animals did not display measurable erythema at any skin testing site. Maximum erythema and average induration were not significantly increased in the NMI:NMI ∆*dot/icm* group compared to negative and unsensitized controls.

**Fig. 8:**
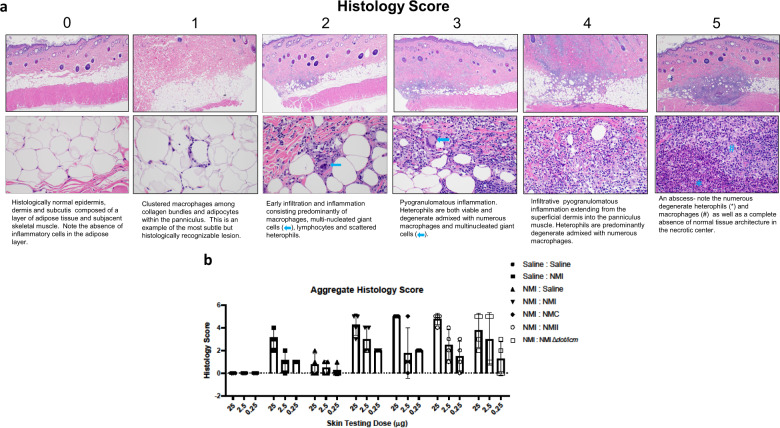
The post-vaccination hypersensitivity response is characterized by guinea pig skin inflammation and abscess formation. **a** Micrographs of skin biopsies from individual skin test sites that were stained by hematoxylin and eosin and scored for lesion severity. Upper and lower micrographs are at 40X and 400X magnification, respectively. **b** Aggregate histology scores are presented as individual scores with mean (bars) and standard deviation (error bars) using the scoring schematic as described in (**a**).

Skin biopsies obtained at 21 days post-skin test were fixed, processed, and stained with hematoxylin and eosin prior to histopathologic evaluation. Skin sections and scoring criteria are described in Fig. 8a. Inflammatory infiltrate was characterized by macrophages (dominant at lower scores) and heterophils (dominant at higher scores). Histological scoring largely corresponded with erythema and induration scores, with all WCV skin tested groups displaying some degree of reactogenicity, regardless of sensitization status (Fig. 8b). All Nine Mile strains yielded similar skin test responses in NMI sensitized animals, with a higher degree of variation among individual responses in the NMI: NMII and NMI:NMI ∆*dot/icm* groups.

## Discussion

Chemically fixed, whole cell *C. burnetii* is a highly efficacious vaccine for the prevention of Q fever in humans^46^ (Q-VAX^®^) and coxiellosis in livestock^47^ (Coxevac^®^). Indeed, Q-VAX^®^ has been an unmitigated success in lowering Q fever cases among at-risk populations in Australia^48^. Despite its remarkable efficacy, several factors limit greater deployment of Q-VAX^®^. First and foremost, vaccination requires careful pre-vaccination serology and skin testing to identify individuals with pre-existing immunity in order to avoid a potentially severe DTH response following vaccination. These pre-immune cohorts can be substantial as exhibited in a past vaccination program at the Rocky Mountain Laboratories^49^ (National Institute of Allergy and Infectious Diseases, National Institutes of Health). Among 384 employees who underwent skin testing, 79 were positive, yielding an approximate reactivity rate of 21% among this population of laboratory workers. Cryptic, pre-existing immunity likely reflects a high percentage of asymptomatic/subclinical *C. burnetii* infections which are thought to account for nearly 60% of human exposures^1^. In a nationwide survey of 4437 United States adults, national Q fever seroprevalence was found to be 3.1%^50^. Studies among French^51^ and Japanese^52^ populations yielded similar seroprevalence among the general population. In groups where Q fever is considered an occupational hazard, such as veterinarians, seroprevalence reports range from 13.5%^52^ to 19%^53^. Thus, pre-existing immunity prior to vaccination is a highly relevant consideration. Q-VAX^®^ skin testing takes 7 days to complete, further complicating vaccine administration. Second, licensed *C. burnetii* WCVs are made from virulent, phase I strains that are heavily regulated and require biosafety level-3 (BSL-3) laboratory conditions for handling. To address these shortcomings, we explored the contribution of major *C. burnetii* surface structures (i.e., LPS and the T4BSS) in vaccine protection and reactogenicity, and the applicability of a genetically engineered avirulent phase I strain as a WCV.

The *C. burnetii* component(s) responsible for post-vaccination hypersensitivity are unknown. In agreement with previous animal studies^37^, we demonstrate that NMI is more potent than NMII as a WCV; however, both strains, along with NMC, show similar reactogenicity in our guinea pig model. In pre-sensitized animals, the reactogenic response occurs with similar kinetics and magnitude regardless of Nine Mile LPS variant utilized in the skin test. Indeed, the original description of the intradermal skin test in humans mentioned that fixed phase II organisms were satisfactory as skin test antigen^54^. Given isogenic NMI and NMII differ only in production of smooth and rough LPS, respectively^44,55,56^, our data indicate LPS *O*-antigen contributes substantially to *C. burnetii* WCV potency while having little influence on the post-vaccination hypersensitivity response.

Consistent with our results, protein(s) or lipid-protein complex(es) have been implicated in the reactogenic response^57^. Soon after development of a hypersensitivity skin test in animals, researchers tested chemical fractions of *C. burnetii* for both vaccine-mediated protection and reactogenicity. Particular insight was provided by examination of trichloroacetic acid (TCA) extracts which were shown to contain the phase I antigen^58^. Extracts displayed 10% and 1% the potency of whole cells as vaccine and skin test antigens in rabbits, respectively^59^. The quantitative differences in protection and reactivity suggested the activities were mediated by distinct antigens and/or immunologic processes. Subsequent removal of protein from TCA extract by phenol treatment eliminated the remaining reactogenicity exhibited by the original extract^57^. Ultimately, it was shown that the phase I antigen extracted by TCA is LPS^58,60^. Indeed, TCA extract was tested as an experimental human “chemovaccine”^61,62^ and a lack of infections among the study cohort suggested efficacy^49^.

Collectively, our data demonstrate that the T4BSS is required for *C. burnetii* pathogenesis, dispensable for vaccine-induced immunity, and may be involved in the post-vaccination hypersensitivity response in guinea pigs. In contrast to highly virulent NMI, NMI ∆*dot/icm* did not cause fever, body weight loss, or splenomegaly in a guinea pig infection model, all indicators of productive infection. Indeed, the NMI ∆*dot/icm* strain appeared to be completely avirulent with no statistical deviation from the saline-treated animals in any of the clinically relevant endpoints we tested. The avirulence of the NMI ∆*dot/icm* mutant was not unexpected as studies have shown that strains with individual *dot/icm* mutations exhibit pronounced intracellular growth defects in vitro^19,20^, in *Galleria mellonella* larvae^22^, and in an immunodeficient SCID mouse model^23^. In our guinea pig infection model, NMI ∆*dot/icm* appears more attenuated than NMII which caused high but transient fever in a single animal and mean weight loss across the experimental group. Greater attenuation of NMI ∆*dot/icm* versus NMII may reflect the fact that NMI ∆*dot/icm* cannot produce a vacuole that supports intracellular growth^20^ while NMII can because of the presence of a functional T4BSS. Thus, more rounds of NMII replication might be expected before the organism is cleared by the host immune system.

On a per microgram level, the NMI ∆*dot/icm* mutant has the same vaccine potency as Q-VAX^®^ or NMI in terms of fever response, body weight change, and splenomegaly following challenge. Additionally, outgrowth of *C. burnetii* following inoculation of axenic media with spleen homogenate indicates WCV-vaccinated animals generally lack viable bacteria in the spleen following challenge, in stark contrast to Saline:NMI unvaccinated control animals. Thus, by 14 days post-infection, bacterial clearance occurs in our guinea pig model of Q fever vaccination.

The vaccine potency of NMI ∆*dot/icm* is notable for several reasons. First, it shows the T4BSS, a macromolecular cell envelope complex, is dispensable for vaccine-induced protection in a guinea pig model of vaccination and challenge. Second, it illustrates that avirulent phase I strains can be genetically engineered and confer protection equivalent to virulent, naturally occurring phase I strains as tested in a guinea pig model. Current methodology used to generate *C. burnetii* mutants requires several passages under positive selection to derive a clonal mutant. Starting with homogeneous phase I NMI, generation of NMI ∆*dot/icm* resulted in a subpopulation of organisms producing phase II LPS as detected by silver staining, a phenomenon we have previously described^30^. However, despite the presence of some phase II bacteria in the NMI and NMI ∆*dot/icm* WCV stocks used in this study, both vaccines performed as well as Q-VAX^®^. These results are in contrast to the weak protection conferred by NMII WCV, which was also reported to be less protective than NMI WCV in a murine vaccination-challenge model^37^. We are currently evaluating several methods to eliminate phase II bacteria generated by serial passage.

NMI ∆*dot/icm*-skin tested animals appeared to experience less severe reactogenic response trends than animals skin tested with wild-type NMI. For example, erythema was not observed at 0.25 μg skin test sites, overall induration was less severe throughout the post-skin testing monitoring period, and histology scores were generally lower. Moreover, 2/4 NMI ∆*dot/icm* skin tested animals showed no measurable erythema at the 25 μg site while all NMI tested animals exhibited measurable erythema. NMI:NMI and NMI:NMC groups exhibited statistically significant increases in maximum erythema compared to Saline:Saline controls and in average induration at 25 μg sites compared to Saline:NMI controls. NMI:NMI, NMI:NMC, and NMI:NMII groups exhibited statistically significant increases in average induration at 25 μg sites compared to Saline:Saline controls. These metrics were not differentiated statically in NMI:NMI ∆*dot/icm* animals, emphasizing the divergent responses in this group compared to other Nine Mile skin tested groups. Consistent with potentially reduced reactivity, the *C. burnetii* T4BSS apparatus has several predicted outer membrane lipoproteins (e.g., DotC, DotD, and IcmN)^17,63,64^, and bacterial lipoproteins are known to modulate DTH responses^65^. Animals that were not sensitized but skin tested with *C. burnetii* (Saline:NMI) displayed relatively low reactivity following skin testing. Due to the absence of prior sensitization, these responses may be considered more irritant or innate in nature^66^. This “baseline” irritant response, particularly evidenced at the 25 μg skin test sites, should be considered in the evaluation of the response of all skin tested animals. Additional considerations regarding this model are the depth of skin test injections as variation may influence characteristics of the response and the unblinded nature of induration measurements. Importantly, variation between animals of the same group and among endpoints in this model indicates a complex biological response possibly related to the use of outbread animals.

Although skin testing with any given dose of NMI WCV did not induce any gross systemic alterations or signs of toxicity, prolonged splenomegaly was observed in several experimental groups. Infected animals displayed significant splenomegaly at euthanasia (63 days post initial inoculation) compared to uninfected animals, and the magnitude of this response did not appear to be influenced by skin testing. Prolonged splenomegaly in a guinea pig Q fever model may be suggestive of continuous host immune system activity and/or bacterial persistence.

Another intriguing observation is the significant decrease in mLN cellularity in NMI ∆*dot/icm*-vaccinated, NMI-challenged animals compared to Saline-mock vaccinated, NMI-challenged animals at 14 days post-challenge This trend was observed for all vaccinated and challenged groups, although not statistically significant. This observation may be related to a difference in the kinetics and/or nature of the immune response during primary infection compared to challenge of vaccinated animals^67^. Although the peak functional T-cell response was likely completed by this time point, along with the decrease in mLN cellularity, NMI ∆*dot/icm*-vaccinated, NMI-challenged animals displayed significantly decreased mLN CD8^+^ T-cell mean flouresence intensity (MFI) compared to Saline-mock infected, NMI-challenged animals. Again, this suggests possible heterogeneity in primary and secondary immune responses. Trends in mLN CD8^+^ and CD4^+^ T-cell frequency indicate potential alterations in these cell populations following both primary infection and vaccination and challenge. The CD8^+^ population appears to be decreased while the CD4^+^ population appears to be increased following infection in vaccinated, challenged animals compared to that of unvaccinated, challenged animals. CD4^+^ and CD8^+^ T cells are known to be required for primary clearance of *C. burnetii* in a murine model^68^ and [MHC-II restricted] CD4^+^ T cells are known to be imperative for the secondary protective response in a murine model^69^. These data suggest a potential divergent, temporally-specific role for each cell type in both primary and secondary responses. Generally, vaccination appeared to yield a distinct cellular population profile compared to primary infection. With the expansion of immune analysis into additional tissues and more comprehensive understanding of relevant responses, correlates of protective immunity following WCV vaccination may be identified in this model.

In summary, our data confirm that the T4BSS is essential for *C. burnetii* virulence in an immunocompetent mammal. However, unlike LPS, the T4BSS is dispensable for vaccine-mediated protection and may be involved in the post-vaccination hypersensitivity response. Based on attenuated virulence in guinea pigs, NMII is exempt in the U.S. from regulation as a select agent by CDC-DSAT (https://www.selectagents.gov/exclusions-hhs.html). It is suitable for work at BSL-2 and is considered non-revertible to full virulence due to deletion of numerous LPS biosynthesis genes. Here, we show that non-revertible NMI ∆*dot/icm* displays virulence attenuation similar to NMII, leading to the possibility that this strain could also be exempted from regulation by DSAT. Exemption and BSL-2 use would make NMI ∆*dot/icm* more widely available to the scientific community to explore its application as an alternative WCV and to examine the role of LPS in *C. burnetii*-host cell interactions. Indeed, innate immune signaling and cellular trafficking are both proposed as LPS-mediated events that differ between phase I and phase II bacteria^27,70,71^. NMI ∆*dot/icm* could also be used as a source of phase I LPS for *O*-antigen-carrier protein conjugate vaccine studies as proposed by Anacker and colleagues in 1963^57^. These studies provide proof of principle that targeted gene deletion can be used to generate alternative WCVs for protection against Q fever and to identify, and potentially eliminate, deleterious DTH antigens.

## Methods

### *Coxiella burnetii* strains

All *C. burnetii* strains were grown in acidified citrate cysteine medium-2 or -D (ACCM-2 or ACCM-D)^72^ at 37 °C, 2.5% O_2_, and 5% CO_2_ and were subsequently stored at −80 °C in cell freezing medium (DMEM with 10% fetal bovine serum and 10% dimethyl sulfoxide). Stocks for vaccination and skin testing (WCVs) were cultured in the same manner as infection stocks, fixed in 4% paraformaldehyde for 12 h, washed twice in sterile PBS, resuspended in USP-grade saline, and stored at −80 °C until vaccination or skin testing. All manipulations of phase I *C. burnetii* stocks and infected animal tissue were performed in a BSL-3 laboratory in accordance with standard operating procedures approved by the Rocky Mountain Laboratories Institutional Biosafety Committee.

### Construction of NMI ∆*dot/icm*

The entire 32.5 kb *dot/icm* locus of the *C. burnetii* NMI strain was deleted using Cre-*lox*. Specifically, the 5′ flanking region of *icmX* (*cbu1652*) was amplified from gDNA by PCR with DotIcm5′-pUC19-Kan-loxP-sacB-F and DotIcm5’-pUC19-Kan-LoxP-sacB-R oligonucleotides. The resulting PCR product was cloned into AvaI digested pUC19-Kan-LoxP-sacB by In-Fusion to create pUC19-Kan-loxP-sacB::DotIcm5′flank. The 3′ flanking region from *icmB* (*cbu1622*) was amplified from gDNA by PCR with DotIcm3′-pJC-CAT-loxP-F and DotIcm3′-pJC-CAT-loxP-R oligonucleotides. The resulting PCR product was cloned into BamHI/SalI digested pJC-CAT-LoxP by In-Fusion to create pJC-CAT-LoxP::DotIcm3′flank. Three successive transformations were carried out with pUC19-Kan-loxP-sacB::DotIcm5′flank, pJC-CAT-LoxP::DotIcm3′flank, and pUC19::*1169*^*P*^-*cre*, using kanamycin, chloramphenicol, and sucrose selection, respectively. The *dot/icm* deletion was confirmed by PCR^30^.

Due to the presence of background wild-type NMI, a *lysCA* selection cassette was inserted into the LoxP site of uncloned NMI ∆*dot/icm* by Cre-*lox* recombination. Briefly, LoxP-P1169-lysCA-LoxP was amplified from pJC-CAT::1169P-lysCA by PCR using LysCAcass-F and LysCAcass-R. The LoxP-P1169-*lysCA*-LoxP PCR was co-transformed into the uncloned NMI ∆*dot/icm* with pUC19::*1169*^*P*^-*cre*. Selection of transformants was carried out using ACCM-D minus lysine. Clonal NMI ∆*dot/icm* was obtained by plating on ACCM-D minus lysine agarose plates incubated at 37 °C, 2.5% O_2_, and 5% CO_2_. The presence of cloned NMI ∆*dot/icm* was confirmed by PCR as previously described^30^. The strain was cultivated and stored as described above. Oligonucleotide primers and plasmids used in this study are listed in Table 2.

**Table 2:** Bacterial strains and plasmids used in this study.

Strain or plasmid	Genotype and/or phenotype	Source
Strains		
*E. coli* Stellar cells	F–, *endA1*, *supE44*, thi-1, *recA1*, *relA1*, *gyrA96*, *phoA*, Φ80∆ *lacZ*Δ *M15*, Δ (*lacZYA* - *argF*) U169, Δ(*mrr* - *hsdRMS* - *mcrBC*), Δ*mcrA*, λ–	Clontech
*C. burnetii* Nine Mile I	NMI, RSA493, phase I, clone 7	Davis et al.^82^ and Seshadri, et al.^83^
*C. burnetii* Nine Mile II	NMII, RSA439, phase II, clone 4	Millar et al.^56^
*C. burnetii* Nine Mile Crazy	NMC, RSA514	Hackstadt et al.^28^
*C. burnetii* NMI ∆*dot/icm*	NMI containing a 32.5 kb *dot/icm* (*cbu1622-1652*) deletion	This study
Plasmids		
pJC-CAT-loxP	pJB2581 containing *cat* driven by *1169* ^*P*^; Cm^r^	Beare et al.^84^
pUC19-Kan-loxP-sacB	pUC19 containing a *1169P-Kan-loxP-sacB* cassette; Kan^r^	Beare et al.^84^
pJC-CAT::1169^P^-lysCA	*1169*^*P*^*-lysCA* cassette cloned into pJC-CAT; Cm^r^	Beare et al.^30^
pUC19::1169^P^-cre	pUC19 containing *cre* driven by *1169* *P*; Amp^r^	Beare et al.^84^
pJC-CAT-loxP::DotIcm3′flank	3′ flanking DNA from CBU1622 cloned into pJC-CAT-loxP; Cm^r^	This study
pUC19-Kan-loxP-sacB::DotIcm5′flank	5′ flanking DNA from CBU1652 cloned into pJC-CAT-loxP; Kan^r^	This study

### Visualization of *C. burnetii* LPS and bacterial enumeration

*C. burnetii* LPS was extracted by modified hot phenol extraction and visualized by silver stain and immunoblot^30,43^. All samples utilized in silver stains and immunoblots were derived from the indicated experiments and were run in parallel. Uncropped gel and blot images are presented in Supplementary Figs. 9 and 10, respectively. The BioRad Precision Plus Protein Dual Color Standard was utilized as a molecular weight marker in these experiments. *C. burnetii* stocks used for infection were quantified using qPCR to enumerate genome equivalents (GE)^43^. Fixed *C. burnetii* used in WCVs were enumerated using a direct bacterial count as modified from Ormsbee et al.^73^. Bacteria were stained with Hoechst dye for 20 min, washed in PBS, then sonicated for 5 min in a bath sonicator (60 sonics/min) to eliminate clumping. Dilutions of bacterial suspensions were imaged on glass slides in triplicate using a Nikkon Eclipse T2 microscope. Bacteria were delinated in micrographs using Cell Profiler™ software and enumerated based on the following conversion factors: average particle count per field × 1705 (# of fields per view) × dilution factor. For WCV dose approximation, bacterial numbers were converted to mg dosages using the purified dry weight calculations of Ormsbee et al.^73^ (3.78 × 10^10^ *C. burnetii* per mg).

### Guinea pigs

Table 3 Hartley guinea pigs were obtained from Charles River (strain code 051) at 4 to 6 weeks of age. Animals were acclimated for at least a week prior to experimental manipulation. In light of historical Q fever virulence studies^29,74^, and to minimize potentially confounding sex-associated factors (e.g., behavior, body weight, hormonal effects), female guinea pigs were utilized in these studies. Animals were housed in individually ventilated plastic cages (Allentown; two animals per cage) with hardwood Sani-chip bedding (PJ Murphy). A high fiber guinea pig diet (Envigo global high fiber guinea pig diet; Teklad, cat n. 2041) and chlorinated, reverse osmosis filtered tap water were administered *ad libitum*. A 12-h light–dark cycle was maintained in animal housing facilities which were kept at 68–72 °F and 40–60% relative humidity with a 50% set point. For all experiments, group numbers equaled 4 animals with the exception of the Saline:Saline group in experiment 2 which only had 3 animals. All animals were housed in approved animal biosafety level 3 (ABSL-3) facilities and manipulated under ABSL-3 standard operating procedures approved by the Rocky Mountain Laboratories Institutional Biosafety Committee and an Institutional Animal Care and Use Committee-approved protocol (ASP 2018-002-E). Animal experiments and procedures were performed in an Association for Assessment and Accreditation of Laboratory Animal Care-accredited National Institutes of Health-National Institute of Allergy and Infectious Diseases animal facility.

**Table 3:** Oligonucleotide primers used in this study.

Primer	Sequence (5*’* to 3*’*)
Primers used to create NMI ∆*dot/icm*	
DotIcm5′-pUC19-Kan-loxP-sacB-F	ACGTTTGCGGCCGCCTGCAGCATCGGCTCAACGACCATCC
DotIcm5′-pUC19-Kan-loxP-sacB-R	AGGCCTTGCAGGCCCTGCAGGTTGACCGATATAATCATCGGACATC
DotIcm3′-pJC-CAT-loxP-F	ACGAAGTTATGTCGACGGTCAGGGATAGTCCCGCTTC
DotIcm3′-pJC-CAT-loxP-R	CGGTACCCGGGGATCCGATACCGATCAGGTGCTCGGG
LysCAcass-F	GCTCGCGTCGACCGGTGAGCTCGGTACCCGGGGATCC
LysCAcass-R	CATGCGCACCACCGGTGATTAATTAGAGAACCTGTTTGTCGAC
Primers used for analysis of NMI ∆*dot/icm*	
CBU1628-F	ATTCGAAAAATTTTCCTAACTC
CBU1628-R	CAACCCTTCAATCATCAACTTAAC
Dot/Icm-KO-F	TTGCCCAGTGGCATAATAAGCG
Dot/Icm-KO-R	CTTCTTAACTAGAATATTTATCGCCCATG
PCRcontrol-3′-F	CAATAAAAACTAACCCAGAAAGTAATTC
PCRcontrol-3′-R	GCTGCTGCATCCGATTCATC

### *C. burnetii* infection

On the day of infection, animals were sedated by isoflurane inhalation using an anesthetic vaporizer with activated charcoal adsorption filters (VetEquip Inc, cat. n. 901801 and 931401) and an IPTT-300 transponder (BioMedic Data Systems) was implanted subcutaneously above the shoulder of each animal in a longitudinal orientation using a large bore needle. Four guinea pigs per group were infected with 10^6^ GE of *C. burnetii* in USP-grade saline via intraperitoneal injection. Negative control animals were mock infected with USP-grade saline for each experiment. Body weights, body temperatures, and any behavioral/clinical changes were recorded daily following infections. Body temperatures were collected using a DAS-8007-P reader (BioMedic Data Systems) at a consistent daily time and a temperature of ≥39.5 °C was defined as fever^74–76^.

### Vaccination challenge

On the day of vaccination, animals were sedated by isoflurane inhalation and IPTT-300 transponders were implanted as described above. Four guinea pigs per group were vaccinated with 25 μg of Q-VAX^®^ or 4% paraformaldehyde fixed *C. burnetii* in USP-grade saline via subcutaneous upper back injection. Negative control animals were mock vaccinated with USP-grade saline for each experiment. Body weights, body temperatures, and behavioral/clinical changes were recorded daily following vaccination. At 28 days post-vaccination, animals were infected with 10^6^ GE *C. burnetii* as described above.

### Post-infection hypersensitivity skin testing

The guinea pig post-vaccination hypersensitivity model was performed as described by Baeten et al.^77^ with modifications. Four guinea pigs per group were infected with 10^6^ GE of NMI or mock infected with saline as described above. At 42 days following infection, animals were sedated by isoflurane inhalation and skin tested with 25, 2.5, and 0.25 μg of *C. burnetii* WCV in USP-grade saline via intradermal injection at three separate sites on each animal’s back. Negative control animals were mock skin tested with USP-grade saline. Body weights, body temperatures, behavioral/clinical changes, were recorded daily after skin testing. Skin testing sites were shaved one day prior to intradermal inoculation (“skin testing”) and weekly one day prior to skin metric measurement. Erythema diameter was measured on days 7, 14, and 21 post-skin testing using a Mitutoyo digimatic caliper 550-311-20. Induration was measured every two days by visual assessment and palpation and scored in a manner similar to that described by T. Fredriksson and U. Petterson^78^. Animals were euthanized 21 days following skin testing.

### Euthanasia, tissue collection, and processing

For all experiments, guinea pigs were euthanized by intraperitoneal ketamine injection followed by exsanguination via cardiac puncture and induction of pneumothorax. Blood was collected by cardiac puncture using Vacutainer^®^ blood collection tubes and needles (BD). Following euthanasia, two mesenteric lymph nodes (Experiments 2 and 3) and the spleen (all experiments) were excised and placed into tubes containing sterile phosphate-buffered saline (PBS; Gibco, pH: 7.4, cat. n. 10010023). For experiment 3, 10 mm skin biopsies were collected from each skin testing site and placed into 5 mL 10% formalin for a 48 h fixation. Lymph nodes were manually dissociated using the frosted ends of two microscope slides. Spleens were dissociated using disposable 15 mL tissue grinders (VWR International, cat. n. 47732-446). Lymph node and spleen cellularity were determined using a Scepter automated cell counter (Millipore, cat. n. PHCC20060) with size exclusion parameters (6 to 36 μm). Spleen suspensions were stored at −20 °C for subsequent analysis. For bacterial outgrowth quantification from spleen tissues (experiment 2), spleens were homogenized as described above and homogenates were used to inoculate ACCM-2 media as described by Omsland, et al.^79^ with the following modifications. Briefly, 5 uL of spleen homogenate was added to 5 mL of ACCM-2 in T25 flasks. After 7 days of growth, *C.burnetii* DNA was extracted from bacteria contained in 1.4 mL of each ACCM-2 culture using the Qiagen DNeasy Blood and Tissue Kit. Quantification of genome equivalents (GE) in extracted DNA was conducted by qPCR using primers specific for *C. burnetii groel*^43^. The estimated bacterial burden per mg of spleen tissue was calculated based on dilution factors and published data showing *C. burnetii* averages approximately 4 logs of growth in ACCM-2 media over a 7 day incubation period^79^. Ct values over 35 were considered background amplification in this assay.

### Flow cytometry

For experiments 2 and 3, single-cell suspensions from mesenteric lymph nodes and spleens were aliquoted into 96-well U-bottom plates at a minimum density of 10^6^ cells per well. Cells were washed in staining buffer (PBS + 1% bovine serum albumin) and in a cocktail of antibodies specific for guinea pig cell surface antigens, including B cells (clone: MsGp10, fluorophore: unconjugated, BioRad, cat. N. MCA567) with secondary antibody (anti-mouse IgG1, clone: RMG1-1, fluorophore: AF700, BioLegend, cat. N. 406632), CD4 (clone: CT7, fluorophore: RPE, BioRad, cat. n. MCA749PE), and CD8 (clone: CT6, fluorophore: FITC, BioRad, cat. n. MCA752F). Following surface staining, cells were washed in staining buffer and fixed overnight at 4 °C using Cytofix (BD, cat. n. 554655). Following fixation, cells were washed in staining buffer and analyzed on a BD LSR II or FACSymphony flow cytometer using FacsDiva software (BD Biosciences). Data analysis was performed with FlowJo 10.0 software (TreeStar Inc., Ashland, Oregon). A minimum of 20,000 events were captured for each sample. Single-stained compensation controls and fluorescence minus one staining controls were included to help set gating boundaries.

### Histology

Skin biopsies were fixed in 10% Neutral Buffered Formalin for a minimum of 7 days, placed in tissue cassettes and processed with a Sakura VIP-6 Tissue Tek on a 12 h automated schedule using a graded series of ethanol, xylene, and PureAffin. Embedded tissues were sectioned at 5 μm, mounted and dried overnight at 42° C prior to staining with hematoxylin and eosin using established methods. Biopsy specimens were evaluated using an Olympus BX53 microscope.

### Statistical analysis

Statistical analyses were conducted using GraphPad Prism version 7.0 (GraphPad Software, La Jolla, CA, USA) and R version 3.6.3^80^. Statistical evidence for differences in group means or geometric means (for splenomegaly and immunological variables) were assessed using two-sample Welch *t* tests, allowing for unequal variances between groups. For each comparison, we computed Wald-type 95% confidence intervals and describe statistical significance with two-sided *p*-values, where we represent *p*-values in equal to or below 0.05 with a single asterisk (*) and *p*-values equal to or below 0.01 with a double asterisk (**). No adjustment was made for multiple comparisons due to the small sample sizes involved. For splenic bacterial outgrowth data, we utilized the Wilcoxon Rank Sum test after transforming all samples above our qPCR amplification threshold (Ct > 35) to the minimum threshold value. For each comparison, we describe statistical significance with two-sided *p*-values, where we represent *p*-values at equal to or below 0.05 with a single asterisk (*), *p*-values equal to or below 0.01 with a double asterisk (**), and *p*-values equal to or below 0.001 with a triple asterisk (***). We used weight measurements of animals in the saline mock-infection control groups in experiments 1 and 2 to estimate separate baseline weight trajectories in the absence of intervention for treated animals in each of those experiments. We fit a linear regression model adjusted for baseline (i.e., pre-treatment) weight using generalized estimating equations with first-order autoregressive working correlation matrix clustering on animal ID utilizing the “geepack” package in R^81^. The counterfactual no-intervention weight trajectory for each treated animal was predicted by incorporating individual baseline weight into the fitted regression model.

### Reporting summary

Further information on experimental design is available in the Nature Research Reporting Summary linked to this paper.

## Supplementary information

Supplementary Information

Reporting Summary

## Data Availability

The authors confirm that all relevant data are included in the manuscript and Supplementary Information.
