# Supplementary material for: Contributions of lipopolysaccharide and the type IVB secretion system to Coxiella burnetii vaccine efficacy and reactogenicity
Source: NPJ Vaccines. 2021 Mar 19;6:38. doi: 10.1038/s41541-021-00296-6 (PMC7979919; doi:10.1038/s41541-021-00296-6)
Supplement: Supplementary file 1 — Supplementary Information [file 41541_2021_296_MOESM1_ESM.pdf]

# Supplementary Files

## Figures

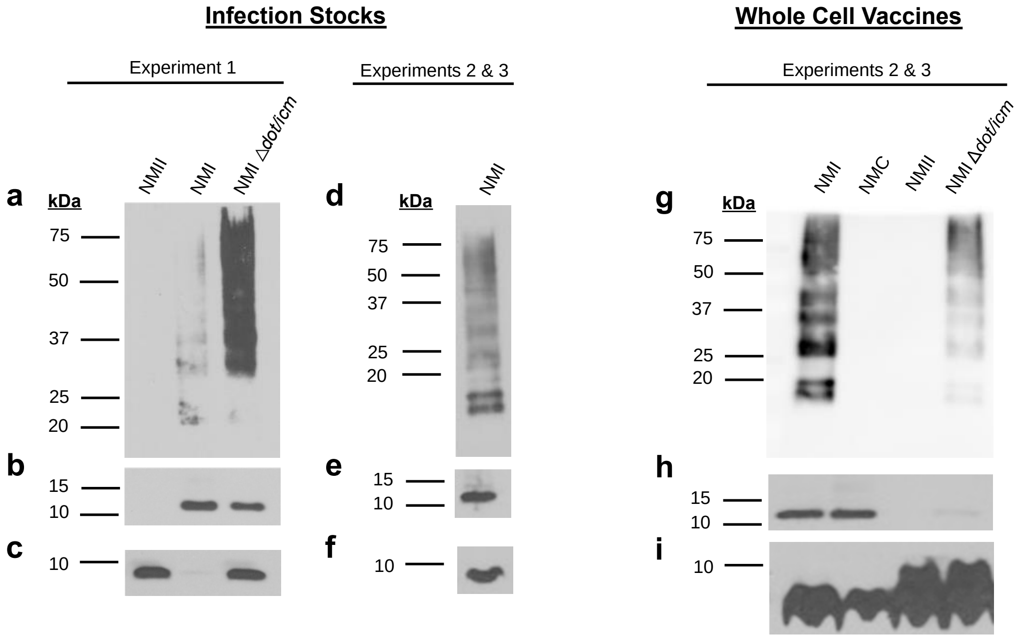


**Supplementary Figure 1. Immunoblot analysis of LPS extracted from *C. burnetii* strains used in this study.**

The LPS of each experimental strain was examined by immunoblot with LPS-specific antibodies. Immunoblots were visualized by imaging of chemiluminescent blots by exposure to film **a-f** and **h**, **i** or on an Azure 600 imaging system **g**. Live infection stocks used in experiments 1 **a-c** and 2 & 3 **d-f** along with whole cell vaccine stocks used in experiments 2 & 3 **g-i** are displayed. Immunoblot reactivity corresponding to phase I **a, d, g**, intermediate **b, e, h**, and phase II **c, f, i** LPS is indicated. Molecular weight markers (kDa) are shown.

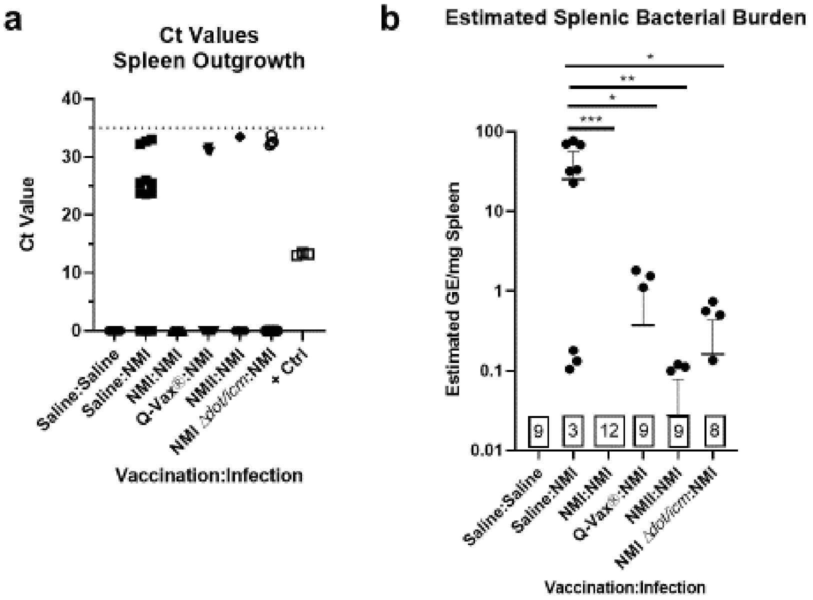


**Supplementary Fig. 2. Estimated Splenic *C. burnetii* burden following WCV vaccination and challenge**

Splenic bacterial burdens were estimated via axenic outgrowth experiments. ACCM-2 flasks inoculated with spleen homogenate were harvested at 7 days post inoculation and qPCR for *C. burnetii* GE was performed. **a** Depicts cycle threshold (Ct) values from ACCM-2 cultures. Ct values over 35 were considered background amplification. Samples showing no amplification were plotted as 0. A positive control (+ ctrl) flask inoculated with NMI stock was included in this experiment. **b** Depicts estimated splenic bacterial burden from each animal (GE/mg spleen). Boxed numbers on the x-axis indicate the number of samples exhibiting no qPCR amplification. Horizontal lines indicate group means. Three Saline:NMI animals (triplicate samples) showed detectable *C. burnetti* (range: ~0.1 - 77 GE/mg) while no animals in the NMI:NMI group showed *C. burneti* outgrowth. QVax:NMI, NMII:NMI, and NMI Δ*dot/icm*:NMI showed 1 animal each with detectable *C. burnetii* (~0.1-1.8 GE/mg). One triplicate sample of an additional NMI Δ*dot/icm*:NMI culture showed detectable bacterial numbers. $*p \leq 0.05$, $**p \leq 0.01$, $***p \leq 0.001$ compared to Saline:NMI values.

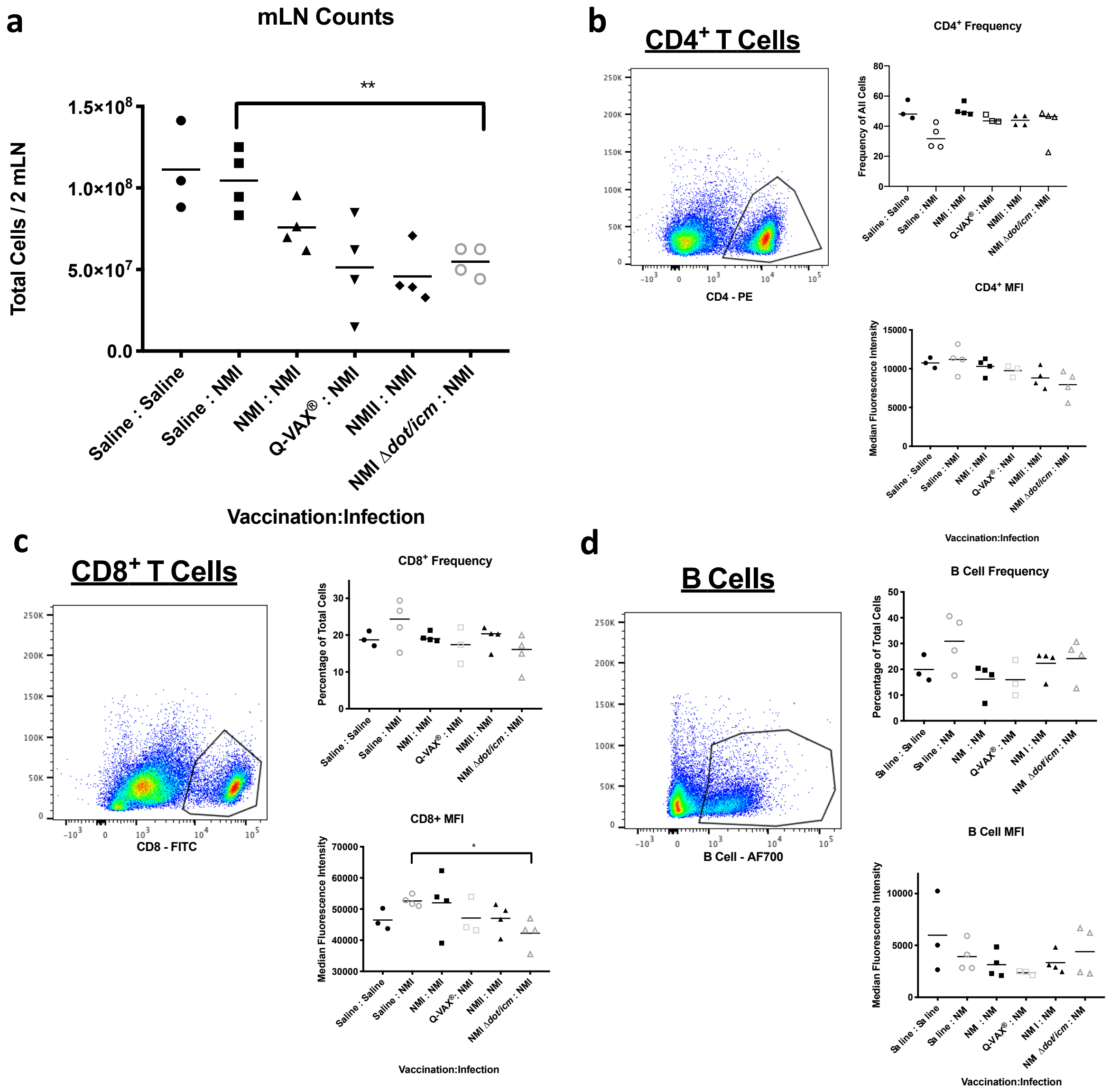


**Supplementary Fig. 3. Alterations of mesenteric lymph node lymphocyte populations in response to primary infection and post-vaccination clearance.**

**a** The cellularity of two mesenteric lymph nodes was determined following cellular suspension. The gating strategy utilized for flow cytometric analysis of each population, cellular frequency, and mean fluorescence intensity are described for $CD4^+$ T cells **b**, $CD8^+$ T cells **c**, and B cells **d**. mLN cellularity, cell frequency, and MFI were evaluated based on differences in group geometric means (horizontal bar). For each reported comparison, p-values were computed for two-sided two-sample t-tests, allowing for unequal variances between groups. No adjustment was made for multiple comparisons due to the sample sizes (n=4 except for Saline:Saline with n=3) and the descriptive nature of the study. $*p \leq 0.05$, $**p \leq 0.01$.

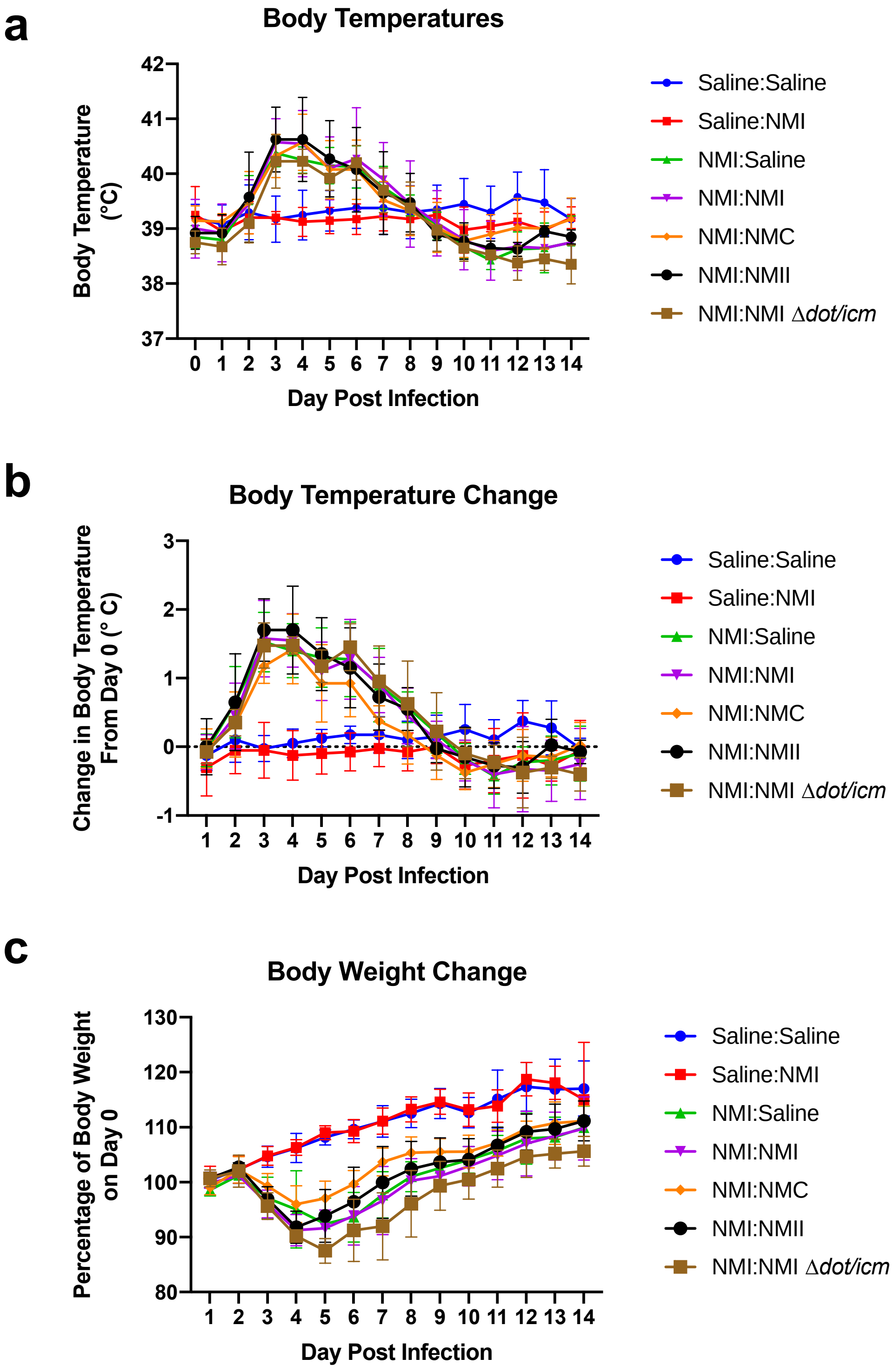


**Supplementary Figure 4. Sensitization of guinea pigs by infection with *C. burnetii* prior to skin testing.**

Body temperature **a**, change in body temperature from day 0 post infection **b**, and percentage of body weight altered compared to day post infection **c** are presented as group means with error bars representing standard error of the mean.

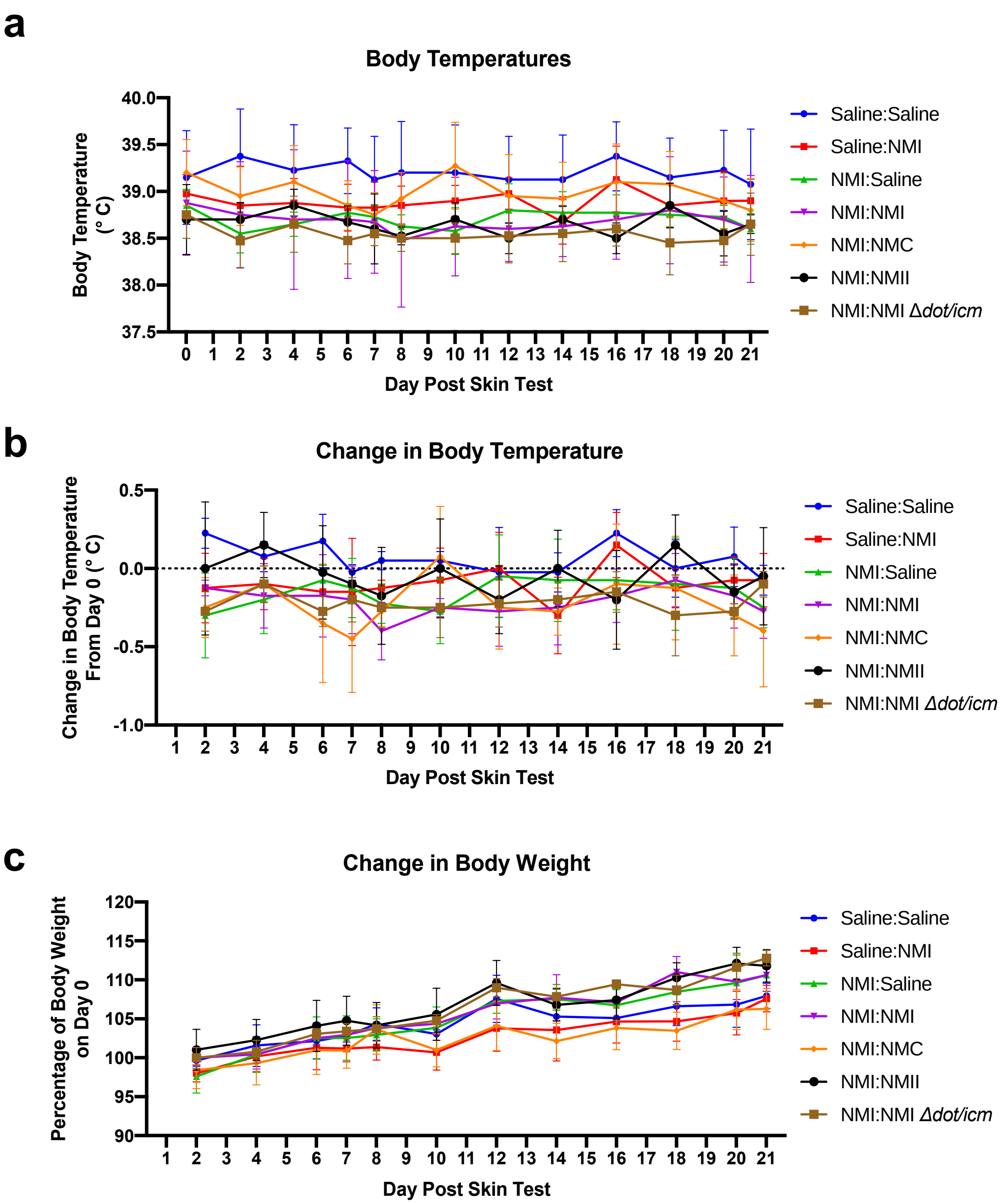


**Supplementary Figure 5. *C. burnetii* skin testing does not induce gross adverse systemic effects in pre-sensitized animals.**

Body temperature **a**, change in body temperature from day post skin testing **b**, and percentage of body weight alteration compared to day 0 post skin testing **c** are presented as group means with error bars representing standard error of the mean.

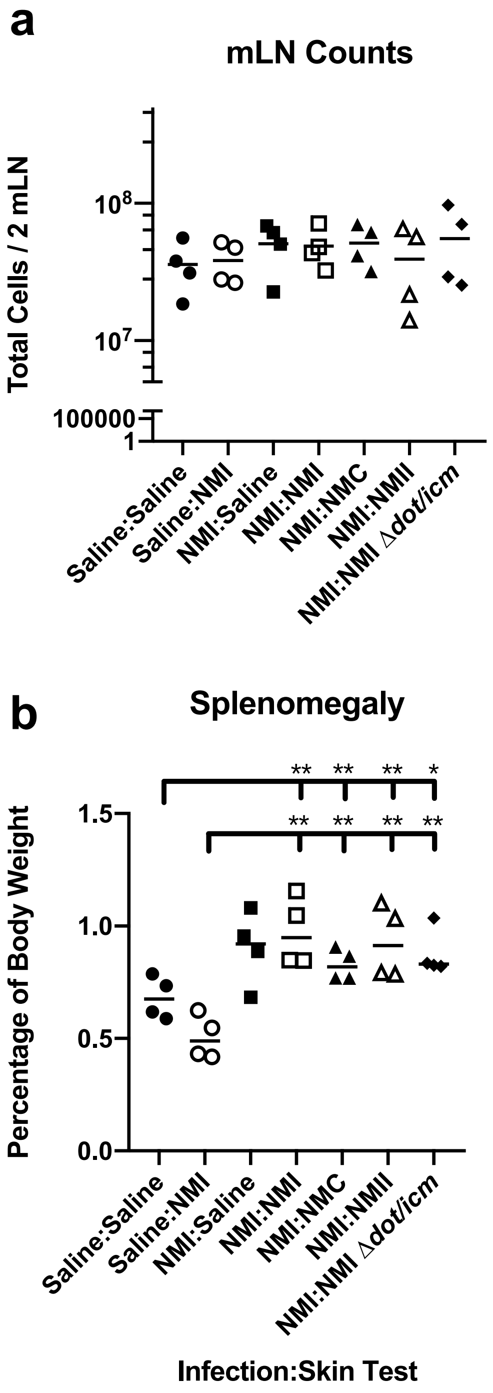


**Supplementary Figure 6. Cellularity of secondary lymphoid organs following *C. burnetii* skin testing**

Mesenteric lymph node (mLN) cellularity (two lymph nodes per animal) is presented in **a**. Splenomegaly is presented as percentage of total body weight in **b**. Both endpoints were evaluated based on differences in group geometric means (horizontal bar) with associated confidence intervals. For each reported comparison, p values were computed for two-sided two-sample t-tests, allowing for unequal variances between groups. No adjustment was made for

multiple comparisons due to the sample sizes (n=4) and the descriptive nature of the study. *$p \leq 0.05$, **$p \leq 0.01$.

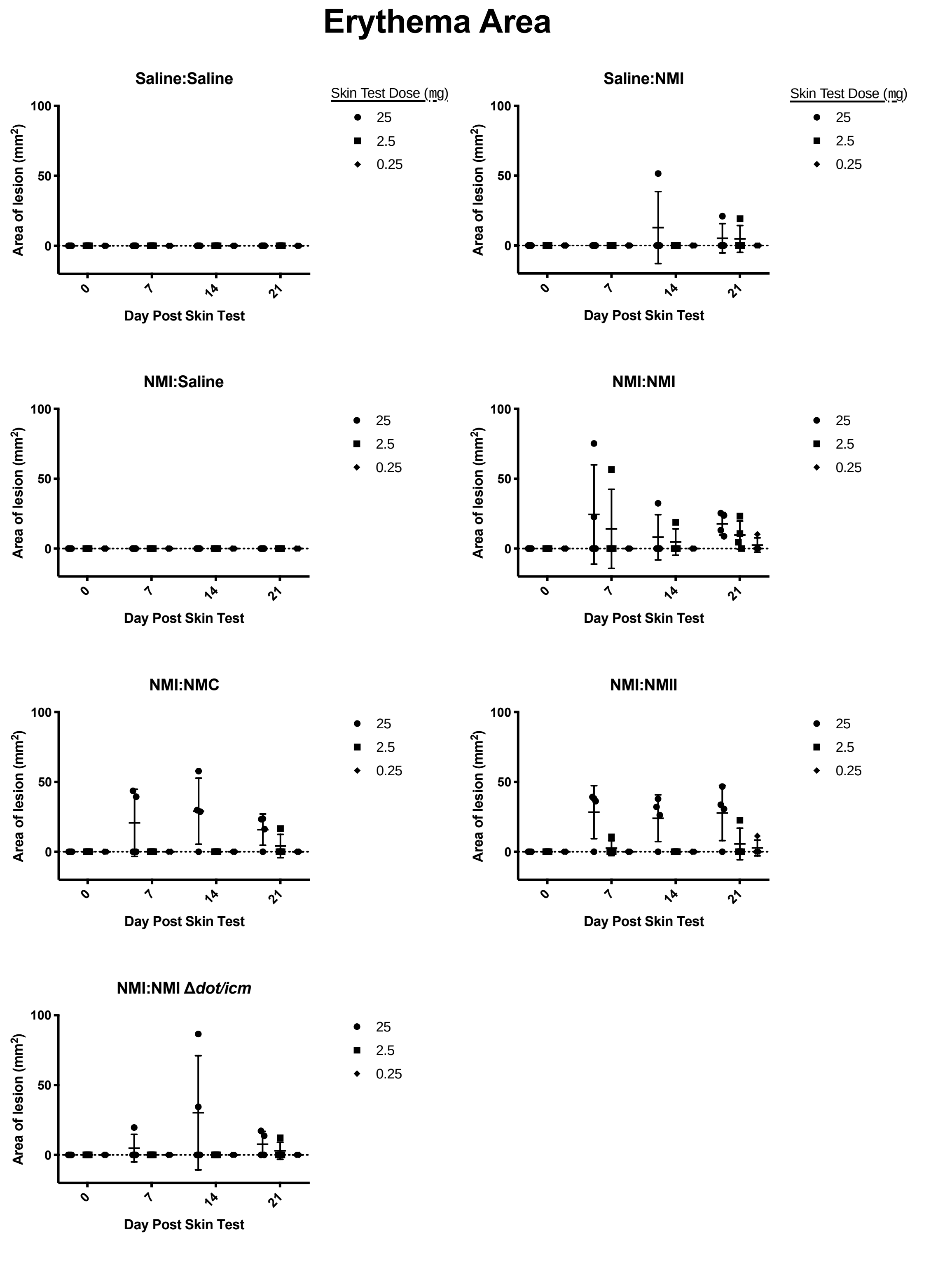


**Supplementary Figure 7. Temporal erythema responses following *C. burnetii* skin testing.** Erythema was measured weekly at each skin testing site and is represented by erythema area ($mm^2$) at any given time point at any given skin testing site.

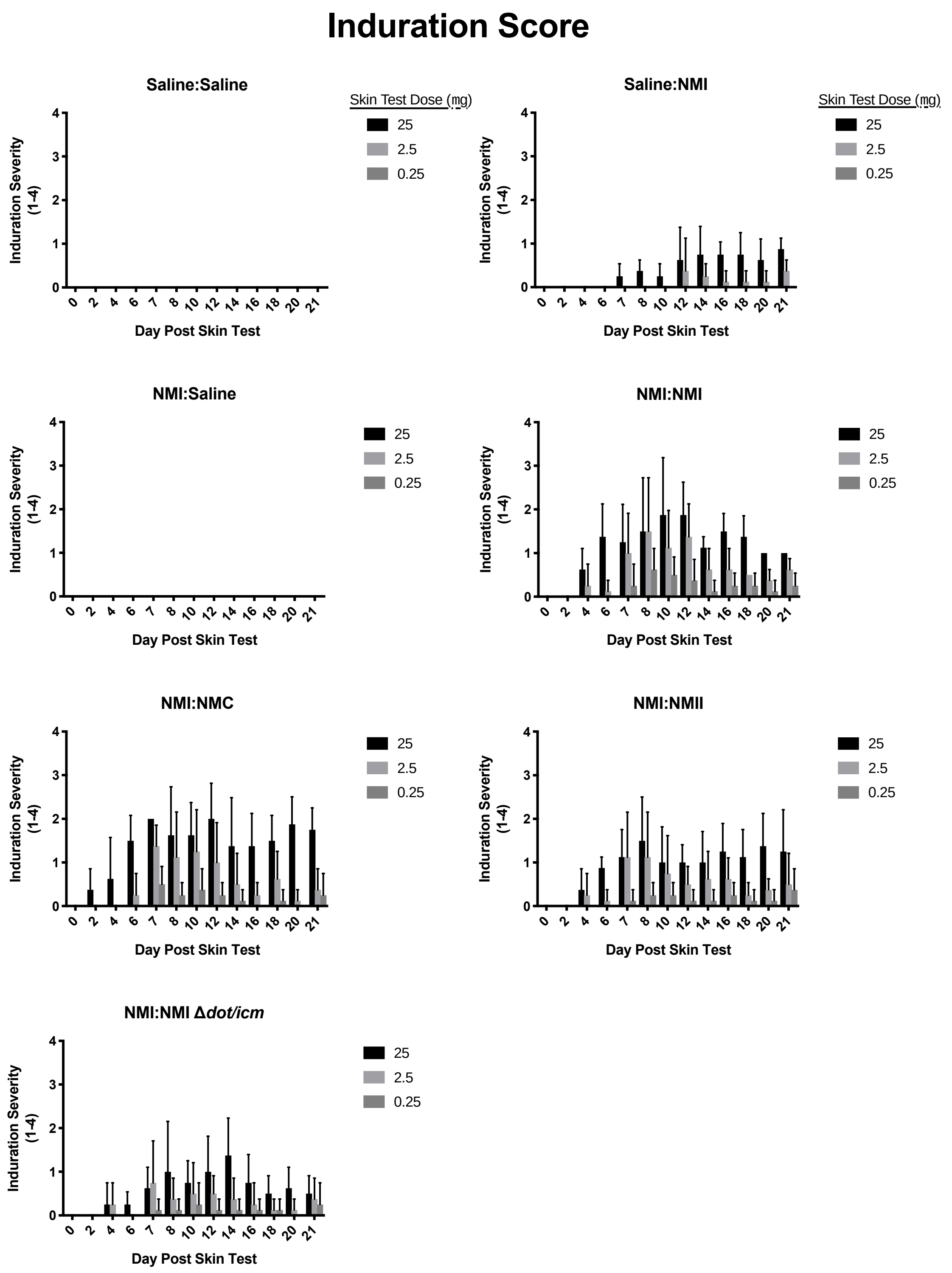


**Supplementary Figure 8. Temporal induration responses following *C. burnetii* skin testing.** Mean induration scores with SEM over the entire 21-day monitoring period for each skin testing site is presented here.

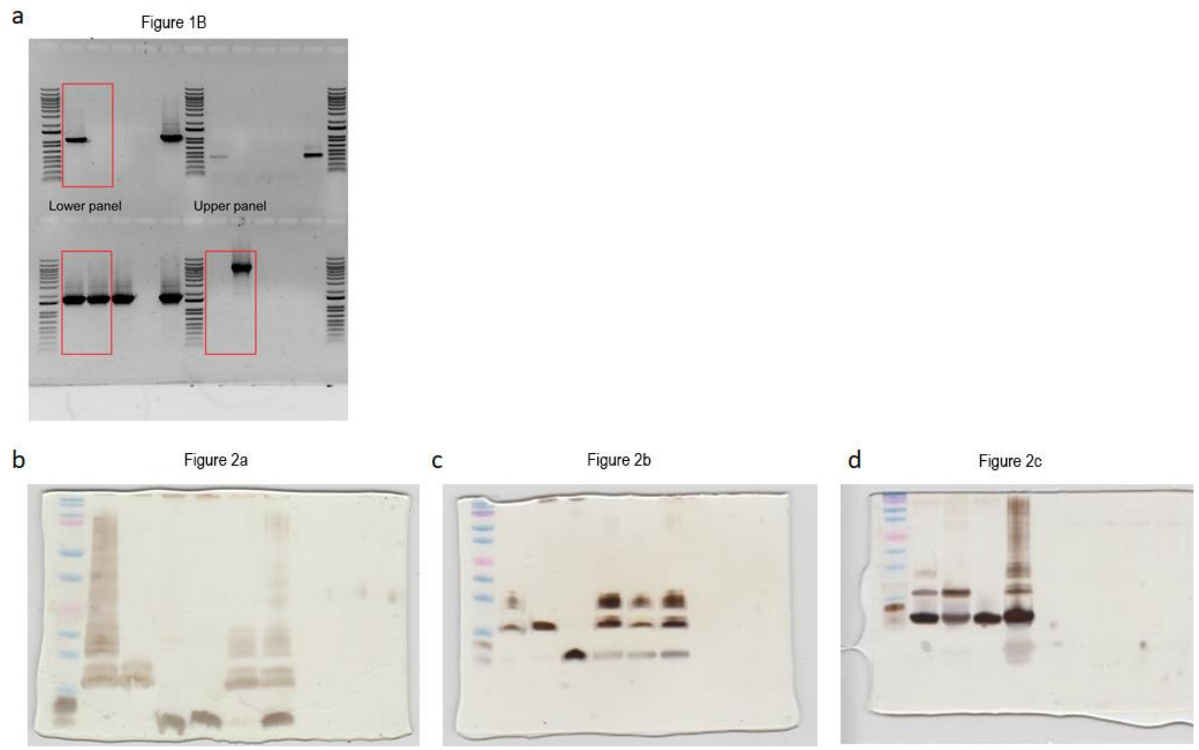


**Supplementary Figure 9. Uncropped Gel Images.** Uncropped PCR gels (a) and silver stains (b-d) pertaining to cropped images shown in Figures 1b and 2a-c, as indicated above. For a, presented lanes are outlined in red boxes and panel placement is indicated with text. For b, lanes 5-7 are presented in Figure 2a. For c, lane 5 is presented in Figure 2b. For d, lanes 2-5 are presented in Figure 2c.

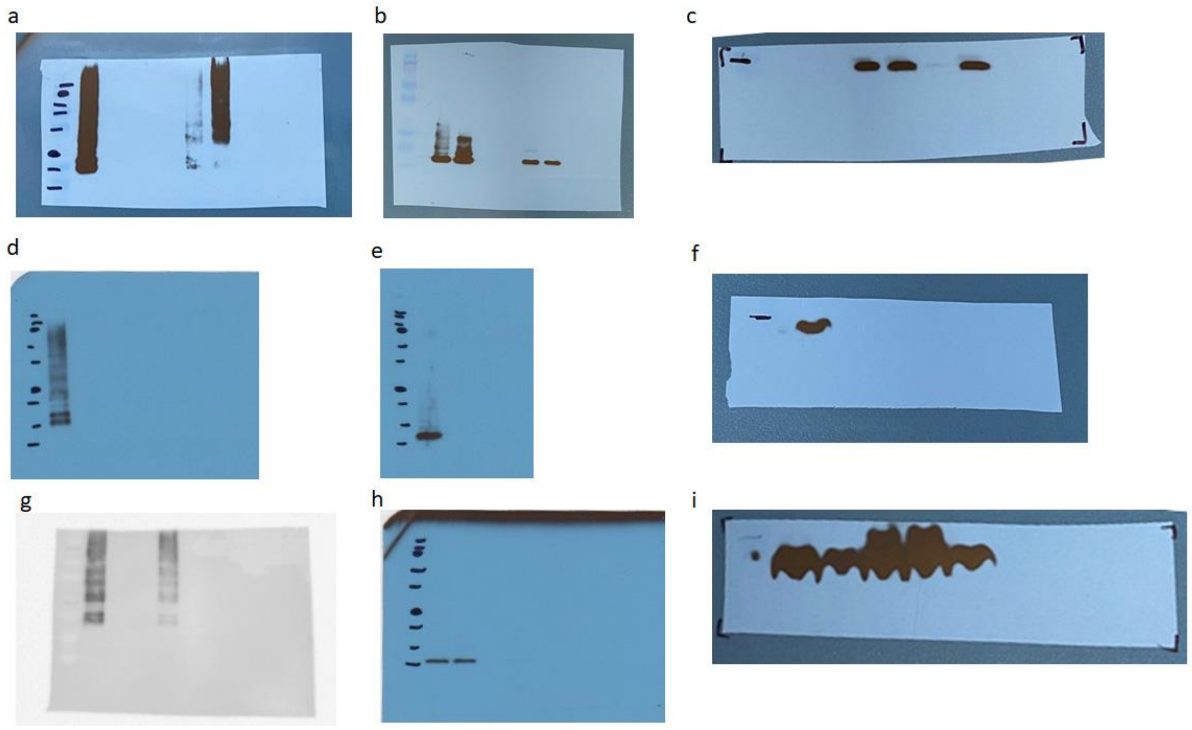


**Supplementary Figure 10. Uncropped Immunoblot Images.** Uncropped immunoblots pertaining to cropped images shown in Supplementary Figure 1, are presented here. A-I labeling corresponds with that of Supplementary Figure 1.
