# Supplementary material for: Contributions of lipopolysaccharide and the type IVB secretion system to Coxiella burnetii vaccine efficacy and reactogenicity
Source: NPJ Vaccines. 2021 Mar 19;6:38. doi: 10.1038/s41541-021-00296-6 (PMC7979919; doi:10.1038/s41541-021-00296-6)
Supplement: Supplementary file 2 — Reporting Summary [file 41541_2021_296_MOESM2_ESM.pdf]

nature research

Corresponding author(s): *Double-blind peer review submissions: write DBPR and your manuscript number here instead of author names.*

Last updated by author(s): *YYYY-MM-DD*

Reporting Summary

Nature Research wishes to improve the reproducibility of the work that we publish. This form provides structure for consistency and transparency in reporting. For further information on Nature Research policies, see Authors & Referees and the Editorial Policy Checklist.

Statistics

For all statistical analyses, confirm that the following items are present in the figure legend, table legend, main text, or Methods section.

n/a	Confirmed	
☐	☒	The exact sample size (*n*) for each experimental group/condition, given as a discrete number and unit of measurement
☐	☒	A statement on whether measurements were taken from distinct samples or whether the same sample was measured repeatedly
☐	☒	The statistical test(s) used AND whether they are one- or two-sided<br>*Only common tests should be described solely by name; describe more complex techniques in the Methods section.*
☐	☒	A description of all covariates tested
☐	☒	A description of any assumptions or corrections, such as tests of normality and adjustment for multiple comparisons
☐	☒	A full description of the statistical parameters including central tendency (e.g. means) or other basic estimates (e.g. regression coefficient) AND variation (e.g. standard deviation) or associated estimates of uncertainty (e.g. confidence intervals)
☐	☒	For null hypothesis testing, the test statistic (e.g. *F*, *t*, *r*) with confidence intervals, effect sizes, degrees of freedom and *P* value noted<br>*Give P values as exact values whenever suitable.*
☒	☐	For Bayesian analysis, information on the choice of priors and Markov chain Monte Carlo settings
☒	☐	For hierarchical and complex designs, identification of the appropriate level for tests and full reporting of outcomes
☒	☐	Estimates of effect sizes (e.g. Cohen's *d*, Pearson's *r*), indicating how they were calculated

Our web collection on statistics for biologists contains articles on many of the points above.

Software and code

Policy information about availability of computer code

Data collection	BD FACSDiva software was used for flow cytometric data collection.
Data analysis	FlowJo version 10 was used to analyze flow cytometric data. Statistical analysis were conducted using GraphPad Prism version 7.0 and R version 3.6.3.

For manuscripts utilizing custom algorithms or software that are central to the research but not yet described in published literature, software must be made available to editors/reviewers. We strongly encourage code deposition in a community repository (e.g. GitHub). See the Nature Research guidelines for submitting code & software for further information.

Data

Policy information about availability of data

All manuscripts must include a data availability statement. This statement should provide the following information, where applicable:

- Accession codes, unique identifiers, or web links for publicly available datasets
- A list of figures that have associated raw data
- A description of any restrictions on data availability

All data are available in the main text or supplementary materials. Additional datasets generated during and/or analyzed in the current study are available from the corresponding author on reasonable request.

Field-specific reporting

Please select the one below that is the best fit for your research. If you are not sure, read the appropriate sections before making your selection.

☒ Life sciences ☐ Behavioural & social sciences ☐ Ecological, evolutionary & environmental sciences

For a reference copy of the document with all sections, see nature.com/documents/nr-reporting-summary-flat.pdf

Life sciences study design

All studies must disclose on these points even when the disclosure is negative.

Sample size	Sample sizes were chosen based on group numbers from prior experiments that exhibited sufficient statistical power for selected endpoints.
Data exclusions	One sample was excluded from flow cytometric analysis (Experiment 2, QVax:NMI group) due to poor sample quality. Criteria were not pre-established.
Replication	Replicates stated in text where appropriate.
Randomization	Experimental animals were randomly assigned into experimental groups based on blinded cage card assignment (experiments 1 and 2) and weight matching prior to treatment (experiment 3).
Blinding	Blinding was not feasible due to rigorous select agent documentation requirements related to animal work.

Reporting for specific materials, systems and methods

We require information from authors about some types of materials, experimental systems and methods used in many studies. Here, indicate whether each material, system or method listed is relevant to your study. If you are not sure if a list item applies to your research, read the appropriate section before selecting a response.

Materials & experimental systems

n/a	Involved in the study	
☐	☒	Antibodies
☒	☐	Eukaryotic cell lines
☒	☐	Palaeontology
☐	☒	Animals and other organisms
☒	☐	Human research participants
☒	☐	Clinical data

Methods

n/a	Involved in the study	
☒	☐	ChIP-seq
☐	☒	Flow cytometry
☒	☐	MRI-based neuroimaging

Antibodies

Antibodies used	AAB-COX-MAB (Bei Resources), 1E4 (a generous gift of Guoguan. Zhang, University of Missouri-Columbia); A6; Anti-Guinea Pig B cell, clone: MsGp10, fluorophore: unconjugated, BioRad, cat. N. MCA567; Anti-mouse IgG1, clone: RMG1-1, fluorophore: AF700, BioLegend, cat. N. 406632; Anti-Guinea Pig CD4, clone: CT7, fluorophore: RPE, BioRad, cat. n. MCA749PE; Anti-Guinea Pig CD, clone: CT6, fluorophore: FITC, BioRad, cat. n. MCA752F
Validation	1E4 antibody was validated by Peng, et al. (Peng Y, Zhang Y, Mitchell WJ, Zhang G. Development of a lipopolysaccharide-targeted peptide mimic vaccine against Q fever. J Immunol. 2012;189(10):4909–20. pmid:23053512; PubMed Central PMCID: PMCPMC3833726); A6 antibody was validated by Beare, et al. (Beare PA, Howe D, Cockrell DC, Heinzen RA. Efficient method of cloning the obligate intracellular bacterium Coxiella burnetii. Appl Environ Microbiol. 2007;73(12):4048–54. pmid:17468273); BioLegend and Bio-Rad flow cytometric antibody validation information can be found on the manufacturers' websites.

Animals and other organisms

Policy information about studies involving animals; ARRIVE guidelines recommended for reporting animal research

Laboratory animals	4 to 6 week old female Hartley guinea pigs were utilized in these studies.
Wild animals	The study did not involve wild animals.
Field-collected samples	Not applicable.
Ethics oversight	All animals were housed in approved animal biosafety level 3 (ABSL-3) facilities and manipulated under ABSL-3 standard operating procedures approved by the Rocky Mountain Laboratories Institutional Biosafety Committee and an Institutional

Animal Care and Use Committee-approved protocol (ASP 2018-002-E). Animal experiments and procedures were performed in an Association for Assessment and Accreditation of Laboratory Animal Care-accredited National Institutes of Health-National Institute of Allergy and Infectious Diseases animal facility.

Note that full information on the approval of the study protocol must also be provided in the manuscript.

Flow Cytometry

Plots

Confirm that:

- [x] The axis labels state the marker and fluorochrome used (e.g. CD4-FITC).
- [x] The axis scales are clearly visible. Include numbers along axes only for bottom left plot of group (a 'group' is an analysis of identical markers).
- [x] All plots are contour plots with outliers or pseudocolor plots.
- [x] A numerical value for number of cells or percentage (with statistics) is provided.

Methodology

Sample preparation	Lymph node cells were processed as described in the Methods section. Briefly, single cell suspensions were stained in staining buffer (PBS + 1% BSA) and fixed overnight in Cytofix.
Instrument	BD LSRII or FACSymphony Cytometers
Software	FlowJo version 10
Cell population abundance	Not applicable
Gating strategy	Singlets (SSC-A/SSC-H) > FSC-A/SSC-A > Gates specified in figures for lymphocyte populations.

- [x] Tick this box to confirm that a figure exemplifying the gating strategy is provided in the Supplementary Information.
